# Mixed messages from benthic microbial communities exposed to nanoparticulate and ionic silver: 3D structure picks up nano-specific effects, while EPS and traditional endpoints indicate a concentration-dependent impact of silver ions

**DOI:** 10.1007/s11356-015-4887-7

**Published:** 2015-06-28

**Authors:** Alexandra Kroll, Marianne Matzke, Marcus Rybicki, Patrick Obert-Rauser, Corinna Burkart, Kerstin Jurkschat, Rudo Verweij, Linn Sgier, Dirk Jungmann, Thomas Backhaus, Claus Svendsen

**Affiliations:** Environmental Toxicology, Eawag, Swiss Federal Institute of Aquatic Science and Technology, 8600 Dübendorf, Switzerland; Ecotoxicology, Acremann Section, NERC Centre for Ecology and Hydrology, Maclean Building, Benson Lane, Crowmarsh Gifford, Wallingford, OX10 8BB UK; Faculty Environmental Sciences, Institute for Hydrobiology, Technical University of Dresden, Zellescher Weg 40, 01217 Dresden, Germany; Department of Materials, Oxford University, Begbroke Science Park, Begbroke Hill, Yarnton, Oxford, OX5 1PF UK; Department of Animal Ecology, Faculty of Earth and Life Sciences, Institute of Ecological Science, Vrije Universiteit Amsterdam, De Boelelaan 1085, 1081 HV Amsterdam, The Netherlands; Department of Biological and Environmental Sciences, University of Gothenburg, Carl Skottsbergs Gata 22 B, 40530 Gothenburg, Sweden

**Keywords:** Biofilms, Periphyton, Silver nanoparticles, Community ecotoxicology, Extracellular polymeric substances (EPS), 3D structure

## Abstract

Silver nanoparticles (AgNP) are currently defined as emerging pollutants in surface water ecosystems. Whether the toxic effects of AgNP towards freshwater organisms are fully explainable by the release of ionic silver (Ag^+^) has not been conclusively elucidated. Long-term effects to benthic microbial communities (periphyton) that provide essential functions in stream ecosystems are unknown. The effects of exposure of periphyton to 2 and 20 μg/L Ag^+^ (AgNO_3_) and AgNP (polyvinylpyrrolidone stabilised) were investigated in artificial indoor streams. The extracellular polymeric substances (EPS) and 3D biofilm structure, biomass, algae species, Ag concentrations in the water phase and bioassociated Ag were analysed. A strong decrease in total Ag was observed within 4 days. Bioassociated Ag was proportional to dissolved Ag indicating a rate limitation by diffusion across the diffusive boundary layer. Two micrograms per liter of AgNO_3_ or AgNP did not induce significant effects despite detectable bioassociation of Ag. The 20-μg/L AgNO_3_ affected green algae and diatom communities, biomass and the ratio of polysaccharides to proteins in EPS. The 20-μg/L AgNO_3_ and AgNP decreased biofilm volume to about 50 %, while the decrease of biomass was lower in 20 μg/L AgNP samples than the 20-μg/L AgNO_3_ indicating a compaction of the NP-exposed biofilms. Roughness coefficients were lower in 20 μg/L AgNP-treated samples. The more traditional endpoints (biomass and diversity) indicated silver ion concentration-dependent effects, while the newly introduced parameters (3D structure and EPS) indicated both silver ion concentration-dependent effects and effects related to the silver species applied.

**Electronic supplementary material:**

The online version of this article (doi:10.1007/s11356-015-4887-7) contains supplementary material, which is available to authorized users.

## Introduction

Natural biofilms in the photic benthic zone are an essential element of stream ecosystems. They are formed by phototrophic and heterotrophic microorganisms, i.e. eukaryotic algae, cyanobacteria, heterotrophic bacteria and fungi. The phototrophic fraction of these communities has initially been referred to as periphyton (Azim et al. 2006). As the terms periphyton and biofilms are now used more commonly to refer to the entire community, we will adopt this terminology in the following. Depending on the type of stream, the communities are the base of the autochthone food web, contribute to oxygen production, serve as a habitat for different life stages of protists and invertebrates and influence stream hydraulics (Lamberti 1996; Nikora et al. 1997). By being a receiving compartment for dissolved substances and for particulates, biofilms alter the stream concentration of pollutants and may release them at a later point of time by detachment/decay of biomass. Biofilm formation is influenced by environmental conditions, such as availability of nutrients, type and roughness of the substratum, as well as hydrodynamic shear force (Azim et al. 2006; Harrison et al. 2006; Labiod et al. 2007; Larned 2010). The species composition of the diatoms inhabiting the biofilms is hence employed in various water quality indices to assess the impact of anthropogenic activities.

The integrity of the biofilms is mediated by species-specific extracellular polymeric substances (EPS) made up of polysaccharides, glycoproteins, low molecular weight acids (LMWA) and neutral/amphiphilic compounds (Bellinger et al. 2010; Flemming and Wingender 2010; Stewart et al. 2013). Important functions provided by EPS are adhesion to surfaces and cohesion to other organisms, the supply of organic matter for the food web, the creation of a matrix for extracellular enzymes, the protection of microorganisms against pathogens as well as the interaction with particles/colloids, metal ions and nutrients (Azim et al. 2006; Flemming and Wingender 2010). Furthermore, EPS may contribute to the colloidal fraction in stream water (Geesey 1982; Tourney and Ngwenya 2014). The exchange of dissolved substances (including nutrients, O_2_) and particles/colloids between periphytic organisms and stream water is thus mediated by EPS. The rate of exchange is limited by the thickness of the diffusive boundary layer (DBL) between the biofilms and the freely flowing water. The DBL is defined by molecular diffusivity being greater than eddy diffusivity (Dade 1993). Its thickness is determined by the flow velocity, viscosity and roughness of the biofilms. The presence or absence and surface and internal structure of the biofilms in turn influence flow velocity and thus deposition of particles and exchange of dissolved compounds (Dade 1993; Nikora et al. 1997, 1998; Salant 2011). Periphyton may be regarded as a special case of a roughness boundary, with properties that constantly change, depending on environmental conditions (Nikora et al. 1998). The latter thus impact the transfer of pollutants—dissolved or particulate—across the DBL and consequently the exposure of the biofilms. In case of a pollutant or a mix of pollutants altering biofilm structure to an extent that roughness and EPS as well as DBL properties are affected, exchange of nutrients, gasses, particles and also exposure to the same or other pollutants will be altered. The effect of anthropogenic stressors on the roughness of periphyton has not been investigated to our knowledge. However, it has been shown that the class of algae (diatoms, filamentous green algae or red algae) that dominates periphyton influences the extent of water velocity attenuation (Dodds and Biggs 2002). EPS composition in heterotrophic biofilms grown from sludge has been shown to shift from polysaccharide-dominated to glycoprotein-dominated with biofilm aging, whereas different flow conditions had no detectable effects (Wagner et al. 2009). Pollutant-induced changes in composition and abundance of EPS of microorganisms and biofilms have not been extensively researched. An exception is research on silver in ionic (Ag^+^) or elemental/nanoparticulate (Ag, AgNP) form, as it is widely used as bactericide. *Escherichia coli* and bacteria in activated sludge showed an increase of EPS production when exposed to sublethal concentrations of AgNP (Joshi et al. 2012; Zhang et al. 2014). Likewise, exposure to AgNP increased the amount of EPS produced in cultures of a marine diatom (Miao et al. 2009). The interaction of EPS with Ag has been suggested to decrease Ag toxicity to microorganisms (Joshi et al. 2012; Kang et al. 2013; Miao et al. 2009); however, Ag may also decrease the activity of extracellular enzymes in periphyton (Gil-Allué et al. 2014). Furthermore, EPS may alter the exposure to Ag fundamentally by reducing Ag^+^ to AgNP as shown for EPS from periphyton and heterotrophic bacteria (Kang et al. 2013; Kroll 2014). The toxic effects of Ag to microorganisms have been reported extensively, especially driven by the increased release of Ag from the photoindustry in the 1970s (Petering 1976). With the increasing use of AgNP and potential release to surface waters, research has focussed mostly on a single species level on the question whether Ag in the form of AgNP exerts different effects than Ag^+^. This question has been evaluated differently depending on the exposure parameters used. In the model algae *Chlamydomonas reinhardtii*, carbonate-stabilised AgNP seemed to decrease growth and photosynthetic activity more than AgNO_3_ when compared to the initially available concentration of Ag^+^ (EC50_5 h_[Photosynthesis], 97.2 μg/L AgNP) (Navarro et al. 2008). A recent study including the model algae *Pseudokirchneriella subcapitata* suggested that toxic effects of citrate-stabilised AgNP between 20 and 80 nm correlated to the concentration of available Ag^+^, whereas AgNP of 10 nm primary particle size showed effects stronger than expected based on [Ag^+^] (Ivask et al. 2014). Currently, two examples of algae communities exposed to AgNP are available: a long-term study in mesocosms after spike exposure to 2.5 mg/L of Ag^+^ and two different types of AgNP (polyvinylpyrrolidone (PVP) and gum arabicum stabilised) (Colman et al. 2014) and a short-term study (2 h) conducted in the dark with Ag^+^ and AgNP (citrate stabilised) (Gil-Allué et al. 2014). The long-term approach showed a decrease of biomass of phytoplankton with no significant differences between the different treatments but also indicated recovery after 6–8 days after spike exposure (Colman et al. 2014). Short-term exposure of periphyton to Ag resulted in Ag^+^-induced decrease in photosynthesis independent of the Ag species applied (Gil-Allué et al. 2014).

Against this background, we analysed the effect of Ag in two different forms, AgNO_3_ and AgNP stabilised with PVP, to benthic biofilms, a receiving compartment for Ag in streams. Benthic biofilms were spike-exposed in artificial indoor streams (AIS) to nominal concentrations of 20 and 2 μg/L Ag and repeatedly sampled over the course of 18 days. We focussed on two parameters that are relevant to the interaction of benthic biofilms with other pollutants and to near-bed hydrology, i.e. EPS composition and 3D structure and related these to biomass, algae species composition, measured Ag concentrations and the amount of bioassociated Ag.

## Material and methods

### Chemicals and nanoparticles

All chemicals were purchased from Fluka, Merck or Sigma-Aldrich if not stated otherwise. AgNP with a nominal primary particle size of 25 nm were produced by wet precipitation from AgNO_3_ in the presence of PVP by NanoSys GmbH (Wolfhalden, Switzerland) as an aqueous suspension with a nominal concentration of 1 g/L (9.27 mM) Ag. The original suspension was kept in the dark.

### Biofilm colonization

Benthic biofilms were collected on August 01, 2013 (d_−11_, Table 1) from Gauernitzbach, a second-order mountain stream of 4.6 km length and a tributary of the River Elbe located northwest of Dresden (Germany; 51° 06′ N, 13° 32′ E (Winkelmann et al. 2008)). The Gauernitzbach has been rented by the Institute of Hydrobiology/TU Dresden for research purposes. Samples were taken in a deciduous woodland valley (mainly alder, maple and oak trees) with an average stream width of 1.2 m and an average slope of 2.7 %. The stream catchment is moderately affected by agricultural run-off (Winkelmann et al. 2008). Biofilms were brushed off stones into the exposure medium (modified Borgmann medium; particle-free and activated carbon-filtered tap water with ingredients; LO4-S and additives E + H (Borgmann 1996), Table S2). The biofilm suspension was transferred to a glass aquarium for settlement on unglazed ceramic tiles (4 × 6 cm) and covered with a 6-cm layer of exposure medium. Phosphorous was added 11 days (d_−11_) and 6 days (d_−6_) prior to test start to reach a final concentration of 0.046 mg/L. Harvesting and transfer of biofilms on tiles (d_−11_) is also described in detail by Rybicki et al. (2012).

**Table 1:** Experimental schedule

Day [d]	−11	−10	−6	−5	−4	−2	0	2	4	7	9	11	14	16	18
Setup															
Biofilm collection	x														
Start AIS	x														
Addition of phosphorous	x		x												
Addition of supplements to AIS		x													
Transfer of tiles to AIS				x											
Addition of NOM					x										
Start of exposure							x								
Biological endpoints															
Silver analysis (bioassociation)						x	x		x			x			x
Taxonomy (light microscopy)							x		x						x
Biomass (POC)							x		x			x			x
Biomass (dry weight)															x
3D structure (CLSM)															x
EPS (LC-OCD-OND)															x
Physicochemical parameters															
EC, pH, O_2_				x		x	x	x	x	x	x	x	x	x	x
SRP-P, nitrogen (N-NO_3_, N-NO_2_, N-NH_4_)				x			x		x			x		x	x
Si, Ca, Mg							x		x			x			x
Cl^−^, F^−^				x			x		x			x		x	x
NOM							x								x
Silver analysis (total/dissolved)						x	x		x			x			x

### Preparation of the artificial indoor streams

Briefly, six equal streams made of stainless steel (4.2 m long and 0.5 m wide) located in a greenhouse with a south–north alignment were used. Details on the design of the AIS are also given in Jungmann et al. (2001) and Licht et al. (2004). Measured intensities and spectra of the irradiation in the greenhouse are available in the Supplementary information (median light levels over 24 h, 3617–3961 lx; Fig. S1). The streams were filled with 450 L activated carbon-filtered tap water up to a level of approximately 10 cm in the flow path. Velocity and cooling devices were launched (d_−11_), the surface water velocity was adjusted to 0.2 m/s and the temperature was maintained at 15 ± 1 °C. Supplements were added to the AIS to generate modified Borgmann medium (d_−10_).

For sampling purposes, three equally long sections in the AIS were defined with zone A being at the inflow, zone B in the middle and zone C at the outflow of each AIS. After biofilms were established (d_−11_–d_−5_) on the ceramic tiles, they were randomly distributed to three separate zones: A (upstream), B (midstream) and C (downstream) in each of the six streams (d_−5_). Each zone consisted of 40 tiles with a total of 120 tiles per stream.

As source for humic acids, organic soil (living garden, DIY-store OBI; Dresden, Germany) was dried until its weight stayed constant (70 °C, UM 400, Memmert); 25 g of dried soil was transferred to tea bags (Celia, Melitta, Germany) and six tea bags were placed in each stream for 24 h for extraction (d_−4_).

### Exposure of periphyton to AgNP and AgNO_3_

AIS were spiked with AgNP and AgNO_3_ on d_0_. A stock solution of AgNO_3_ (≥99, 9 %, p.a., Carl Roth GmbH) containing 1000 mg/L Ag (note that all concentration numbers for both AgNP and AgNO_3_ throughout this paper are based on the mass of Ag) was prepared freshly in double deionised water (−18.2 MΩ, Millipore, USA). The original AgNP suspension contained 82 % of the nominal concentration (7.6 mM) with 0.7 % dissolved Ag, and the AgNO_3_ stock solution contained 90 % of the nominal Ag concentration with 100 % dissolved Ag. AgNP and AgNO_3_ were dosed accordingly to the nominal stock concentrations to a final nominal concentration of 2 and 20 μg/L Ag at the water inflow of the AIS. Exposure concentrations were chosen to be at the lower end of or below the reported short-term effective concentrations of silver in single algae (Bondarenko et al. 2013; Ivask et al. 2014; Pillai et al. 2014), phytoplankton (Colman et al. 2014) and periphyton (Gil-Allué et al. 2014) and one to two orders of magnitude higher than the Ag concentrations reported in situ (Oertel 1991). The treatments are referred to as Ag20 and Ag2 for 20 and 2 μg/L AgNO_3_ and as NP20 and NP2 for 20 and 2 μg/L AgNP. Using one replicate per treatment was the trade-off between available resources and research questions. Both ionic silver and silver nanoparticle exposures in two concentrations were applied to investigate if there are general differences between the two silver species. When working with natural communities, it is important to compare exposures within one experiment because the community structure may hugely vary between experiments. We considered two concentrations per silver species to be more important than replication of one to be able to cover a broader range of potential effects. To be able to judge the variation between different treatments and in the system, two untreated control channels were run.

### Monitoring of physicochemical parameters in the AIS

Conductivity and temperature (LF340, WTW), pH (pH 3110, WTW) and oxygen concentration (Oxi340i, WTW) were measured regularly. The concentration of soluble reactive phosphorus and ammonium (as nitrogen) was determined photometrically according to appropriate guidelines (phosphorus: DIN 38405 D11-; ammonia: DIN 38406 1983). Ammonia concentration was calculated according to Hamm (1991). The concentration of nitrate and nitrite (as nitrogen) as well as chloride, fluoride, and sulphate was determined using anion-exchange chromatography (ISC90, Dionex) with an 8-mM carbonate/1-mM hydrogen carbonate solution as mobile phase and an IonPac AS14A separation column (Dionex) as stationary phase. Limits of quantification (LOQ) were as follows: 120 μg/L F^−^, 150 μg/L Cl^−^, 60 μg/L NO_3_-N, 90 μg/L NO_2_-N and 70 μg/L SO_4_-S. Calcium and magnesium content was quantified by cation-exchange chromatography (930 Compact IC Flex, Metrohm) with a 8-mM HNO_3_/1.197-mM dipicolinic acid solution as mobile phase and a Metrosep C 6—250/4.0 separation column (Metrohm) as stationary phase. Silica was determined colorimetrically based on the reduction of siliciomolybdate to silicomolybdous acid in the presence of ascorbic acid using the Autoanalyzer AA3, Bran+Luebbe (Contrec). Detection limits were as follows: 5 mg/L Ca^2+^, 2.5 mg/L Mg^2+^ and 1 mg/L H_4_SiO_4_. Physicochemical parameters and measured data are summarized in Table S1.

### Modelling of equilibrium speciation with Visual MINTEQ 3.1

The chemical equilibrium model Visual MINTEQ 3.1 (VMINTEQ) was used to model the chemical species distribution in the AIS at the start of the exposure. Concentrations of the compounds used as input (Table S2) were based on the exposure medium composition and mean values of the tap water used (in 2013, measuring site 62-000-102-411 (DREWAG 2013)). Silver was included as total concentration of Ag (20 and 2 μg/L, corresponding to 185 and 18.5 nM). Natural organic matter (NOM) was included according to NICA-Donnan (3 mg/L, according to measured NOM in the AIS, Table S1). The model was run using the standard databases for 15 °C without fixing the pH or ionic strength, without fixed species, finite solids or excluded species.

### Characterisation of nanoparticle dispersions

The nominal exposure concentrations (2 and 20 μg/L) were lower than the detection limits of the standard NP characterisation methods described below. Thus, we analysed PVP-AgNP and AgNO_3_ dispersed in exposure medium at 5, 1 and 0.02 mg/L depending on the subsequent characterisation method. Dispersions of 20 mL were stirred at 400 rpm and 15 °C. Illumination was provided in 12:12 h light/dark cycles by BioSun fluorescent tubes with a radiation similar to the sunlight spectrum (Radium Lampenwerk GmbH, Germany, ML-T8 36W/965/G13B; photon flux during light periods, 100 μE/m^2^ s). pH was constant during exposure. Samples were prepared in triplicates (vessels: SNAP-CAP, Huber Switzerland, 8.9227.08; magnetic stir bars, with PTFE coating, 6 × 20 mm, Huber Switzerland, 13.1120.06). Dispersions contained 0.02 % (*w*/*v*) NaN_3_. In particular, NP hydrodynamic diameter was determined by dynamic light scattering (DLS) and nanoparticle tracking analysis (NTA); zetapotential was derived from electrophoretic mobility (EPM) measurements; light absorption was measured by UV-VIS spectroscopy to identify surface plasmon resonance (SPR).

### Dynamic light scattering and electrophoretic mobility

The hydrodynamic diameter of the NP was derived from DLS measurements and the zetapotential from the EPM, both measured on a Zetasizer Nano ZS (nano ZS, Malvern Instruments). For comparison with data from the literature, the Smoluchowski model was used to calculate the zetapotential from EPM measurements. Three measurements were taken per sample and the autocorrelation function was analysed using the cumulant analysis algorithm resulting in a mean size (*z*-average) and a standard deviation (polydispersity index, PDI). Data sets with a PDI above 0.5 were not taken into account (for settings, see Text S1). DLS measurements performed on aqueous suspension without NPs did not indicate any particles.

### Nanoparticle tracking analysis

NTA (NanoSight LM10 equipped with a LM14 temperature controller, NanoSight Ltd.) was used to determine a number-based particle size distribution. Each sample was directly measured three times for 60 s without any further processing (no dilution). All NTA videos were analysed with the same settings in batch processing mode (for settings, see Text S2). Analyses that resulted in less than 200 tracked particles were not used. Videos were analysed using the NanoSight NTA 2.3 Analytical Software (NanoSight Ltd.).

### UV-VIS absorption

UV-VIS light absorption (190-900 nm) of PVP-AgNP dispersions and AgNO_3_ in exposure medium (5 mg/L and 20 μg/L) was recorded with a UVIKON 930 spectrophotometer (Kontron Instruments). AgNP show size- and surface-specific SPR which results in a specific light absorption spectrum (Evanoff and Chumanov 2005).

### Transmission electron microscopy

Transmission electron microscopy (TEM) was used for a quality check of the stock dispersions regarding the particles size, shape and homogeneity of the dispersion. Experiments were carried out on a JEOL 2010 analytical TEM (JEOL Ltd, Japan), equipped with a LaB6 electron gun and operated between 80 and 200 kV. Samples were dispersed in water and a drop of the dispersion was deposited on a holey carbon-coated copper TEM grid and dried at room temperature for several hours before examination (Matzke et al. 2014).

### Sampling for the analyses of biofilms and fate of Ag in the exposure setup

Table 1 gives an overview of the schedule of the experiment including time points and parameters assessed. Biofilms were analysed for dry weight, EPS composition, 3D structure and green algae, diatom and cyanobacteria taxonomy as well as the amount of bioassociated Ag. For dry weight, EPS composition, taxonomy and Ag quantification, four tiles were randomly picked from each of the three sampling zones. Biomass was sampled by scraping into 40 mL of exposure medium and resuspension by gentle pipetting. The suspensions were split for the different endpoints (see “Quantification of biomass-associated Ag by AAS”, “Estimation of biomass by measurement of particulate organic carbon” and “Taxonomic analysis of phototrophic organisms” sections). For the analysis of 3D structure, tiles from sections A and C were randomly sampled and treated as described in the section “Surface and 3D structure of periphyton”. Samples for inductively coupled plasma mass spectrometry/atomic absorption spectrometry (ICP-MS/AAS) analysis of total and dissolved Ag in the exposure medium were taken according to the schedule (Table 1) and treated as described in the section “Quantification of total and dissolved Ag by ICP-MS”. To obtain time-averaged dissolved Ag concentrations, two diffusive gradients in thin films (DGT) (Davlson and Zhang 1994; Navarro et al. 2008) samplers were deployed per AIS from d_10_ to d_18_. Two additional DGT samplers were deployed in Gauernitzbach for 3 weeks during the same period.

### Quantification of total and dissolved Ag by ICP-MS

Liquid samples were centrifuged to separate particles (>3.5 nm) and dissolved ions (25 °C and 20,800*g* for 3 h (Ag, *ρ*_Ag_ 10.5 g/cm^3^). The supernatants (0.6 mL) were acidified (0.65 % HNO_3_) and stored at 4 °C. Non-centrifuged samples for the determination of total Ag concentration as well as DGT membranes were stored in the same manner. All plastic ware was washed with 1 % HNO_3_ prior to use to remove potential metal contaminations. Two non-deployed DGT samplers and fresh exposure medium were analysed regarding Ag background. All samples were treated by microwave digestion prior to ICP-MS analysis; 0.5 mL of each sample or one DGT membrane was digested with 4 mL of 65 % HNO_3_ and 0.5 mL of 30 % H_2_O_2_ in a microwave digestion unit (MLS ultraClave 4; 10 min at 180 °C/100 bar, 14 min at 210 °C/100 bar) and diluted 1:100 with nanopure water (18.1 MΩ cm, Milli-Q). One sample per run contained only HNO_3_ and H_2_O_2_ to determine the background concentration of Ag. Ag concentrations were measured by HR-ICP-MS (Element 2 High Resolution Sector Field ICP-MS; Thermo Finnigan). The instrument was calibrated with a multi-element mass standard (Merck, 1113550100). The calibration curve for data analysis was made with the calibration standard SCP-33-MS (140-130-321, PlasmaCAL) in the concentration range 0–20 μg/L. A reference with a concentration within the calibration range was measured every 10 samples, and the calibration samples were measured every 40 samples. The LOQ of Ag was 0.01 μg/L. Mean recovery was 96.9 ± 3.7 % for total Ag from AgNP and 96.5 ± 3.4 % for AgNO_3_.

### Quantification of biomass-associated Ag by AAS

An aliquot of the biofilm suspension (see “Sampling for the analyses of biofilms and fate of Ag in the exposure setup”) was spun down at 2000*g* for 30 min (Centrifuge 5810R Eppendorf, Germany, high-speed fixed angle rotor F-34-6-38); the supernatant was removed and the pellet was frozen at −20 °C for sample shipping and final sample preparation for the analysis with a graphite furnace AAS. The biomass was freeze dried for 72 h, and samples were weighed on a super micro balance; 0.5 mL of a mixture of HNO_3_/HClO_4_ (ratio 7:1) was added and the samples were placed on a dry block heater for cell disruption. This step was followed by a dissolution step in 1 M HCl (1 mL) before the samples were measured on a graphite furnace AAS to determine the concentration of the total silver associated to the biomass.

### Estimation of biomass by measurement of particulate organic carbon

A subsample of 2.5 to 10 mL of the periphyton suspension taken at d_0_, d_4_, d_11_ and d_18_ (section “Sampling for the analyses of biofilms and fate of Ag in the exposure setup”), depending on the periphyton biomass, was vacuum filtered (−0.2 bar) over pre-ashed glass fibre filters (500 °C >45 min, MGF, 25 mm diameter, Sartorius) for analysis of particulate organic carbon (POC). Filters were placed in petri dishes after filtration and dried at 70 °C (12–24 h) for analysis of POC. Dried filters were stored in a desiccator until combustion analysis in a carbon analyser (C-200, Leco, USA).

### Taxonomic analysis of phototrophic organisms

Suspended biomass sampled on d_0_, d_4_, d_11_ and d_18_ was fixed with 0.01 % glutaraldehyde and 0.1 % paraformaldehyde (*w*/*v*, stock in tap water) and stored at 4 °C in the dark. Samples were diluted 1:10 three times independently in tap water and 1 mL was transferred to a Uthermol’s chamber for microscopic analysis. An inverted microscope (Zeiss Axiovert 135) was used to identify and count (Zeiss EC Plan-Neofluar ×40/0.75 objective, Zeiss EC Plan-Neofluar ×100 1.3 oil objective, if necessary) algae and cyanobacteria genera and, if possible, species within three fields of vision. Abundance was described as dominant (“5”, >30 of the cell number counted), frequent (“4”, 10–30 %), regular (“3”, 3–10 %), scarce (“2”, 1–3 %) and sporadic (“1”, <1 %) based on two taxonomic references and the recommendations by the Swiss Federal Office for the Environment (Hürlimann 2007; Hustedt 1976; Pascher et al. 1987–1997). Species/genera of the phototrophic organisms were analysed in a semi-quantitative manner in samples at the start of the exposure and after 4 and 18 days of exposure to AgNO_3_, resp. PVP-AgNP (d_0_, d_4_, d_18_). Due to the data being semi-quantitative, we did not perform statistical tests but use qualitative description of the data.

### Extraction and characterisation of extracellular polymeric substances from periphyton and determination of dry weight

EPS were extracted from samples at d_18_ and were analysed for organic carbon and organic nitrogen size distribution and protein content. The extraction procedure was performed as described previously (Kroll 2014; Stewart et al. 2013). The harvested biomass (section “Sampling for the analyses of biofilms and fate of Ag in the exposure setup”) was sonicated in a water bath (Sonorex RK100H, Bandelin) for 30 s. Fine sediment and larger biomass was allowed to settle for approximately 1 min, and the supernatant was removed and centrifuged at 2000*g* for 10 min (Centrifuge 5810R Eppendorf, Germany). Biomass was resuspended a second time in 2 mL/tile fresh solution and treated as described above. The resulting biomass pellets were frozen in liquid nitrogen, stored at −20 °C and subsequently lyophilized to determine dry weight. All supernatants were sequentially filtered (1 μm glass fibre [VWR], 0.45 μm polypropylene [PALL] and 0.22 μm PES [Millipore] filters). Filters were washed with nanopure water (18.1 MΩ cm, Milli-Q) prior to use. EPS extracts were stored in glass bottles at 4 °C (0.02 % (*w*/*v*) NaN_3_). All extraction steps were performed on ice, and the water bath for ultrasound treatment was at room temperature.

Organic carbon (OC) and organic nitrogen (ON) size distribution was measured by size exclusion chromatography-organic carbon detection-organic nitrogen detection (LC-OCD-OND) as described previously (Stewart et al. 2013). Samples were diluted 1:20 with nanopure water (18.1 MΩ cm, Milli-Q) directly before analysis. A size exclusion column (250 × 20 mm, Toyopearl TSK HW-50S) was used to separate EPS compounds. To quantify the carbon background of the extraction protocol, an aliquot of extraction buffer was treated the same way as periphyton suspensions and then assessed by LC-OCD-OND. The mobile phase was phosphate buffer (24 mM, pH 6.6) and the acidification solution was phosphoric acid (60 mM, pH 1.2). The detection limit was 10 μg/L for both OC and ON. Retention times of 35–70 min correspond to 70–0.5 kDa model proteins and 28–0.1 kDa model polysaccharides (Stewart et al. 2013). The software FIFFIKUS was used to quantify total organic carbon (TOC), dissolved organic carbon (DOC) and chromatographable DOC compounds (cDOC). The chromatograms obtained from LC-OCD-OND are integrated to determine the amount of biopolymers (high M_r_ polysaccharides and proteins), humic substances (HS), building blocks of HS, low M_r_ acids and amphiphilic/neutral compounds (alcohols, aldehydes, amino acids and ketones).

### Surface and 3D structure of periphyton

To estimate the surface and internal 3D structure of periphyton, we acquired chlorophyll fluorescence and reflected laser light, and the fluorescence of a green fluorophore (FITC) was coupled to a lectin from *Helix pomatia* (HPA) which binds α-*N*-acetylgalactosamine residues. HPA was selected based on a screening with about 70 different fluorophore-coupled lectins for its capacity to stain algal surfaces, bacteria and the interstitial space which is where EPS are expected (Neu and Lawrence 1999; Webb et al. 2003). We found that in the tested periphyton samples, HPA staining exceeded the staining efficiency of the *Aleuria aurantia* lectin (AAL) using volumetric measurements of EPS in heterotrophic biofilms as established by Staudt et al. (2004). AAL contains five fucose-binding sites that preferentially bind fucose linked (α-1,3, α-1,2, α-1,4 and α-1,6) to *N*-acetyllactosamine (Romano et al. 2011).

Tiles covered with periphyton were sampled from sections A and C from each channel (two tiles per section) on d_18_. The biofilms were fixed with 3 % formaldehyde (*w*/*v* in tap water) at 4 °C in the dark. One 1 × 1 cm patch from the bottom left part of each tile was stained with 50 μL of a 100-μg/mL HPA lectin solution (Sigma Aldrich, L-1034) for 10 min in the dark at room temperature on a glass slide. Biofilms were washed three times with tap water and directly imaged by CLSM (Leica SP5). Five z-stacks (246.03 μm × 246.03 μm × individual height; step size 0.5 μm) were recorded per patch in sequential mode. The 488-nm laser line was used for excitation and chlorophyll *a* (650–750 nm; gain 621, offset 0) and FITC fluorescence (510–550 nm; gain 767, offset 0) and reflected light (483–492 nm; gain 411, offset 0) were detected.

CLSM data was analysed by Imaris x64 7.6.4. Data obtained for chlorophyll *a* and FITC fluorescence and reflected laser light was transformed into a surface (Text S3). The three surfaces were then merged and the information on occupied and void voxels was used to calculate the total occupied volume and the roughness coefficient according to Murga et al. (1995):

1 $$ {R}_{\mathrm{a}}^{*}=\frac{1}{N}{\sum}_{i=1}^N\frac{\left|{L}_{\mathrm{f}i}-{L\bar{\mkern6mu}}_{\mathrm{f}}\right|}{{L\bar{\mkern6mu}}_{\mathrm{f}}} $$

The roughness coefficient is based on the individual thickness measurement *L*_f*i*_, the sample mean thickness *L*_f_ and the number of thickness measurements *N*, and is a measure for the surface heterogeneity of the visualised biofilm. It takes into account the influence of the mean thickness on the previously suggested roughness coefficient (Nowicki 1985). It has recently been used to quantify the removal of heterotrophic biofilms from surfaces based on CLSM imaging of lectin-stained samples (West et al. 2014).

### General data analysis

GraphPad Prism 4 for Windows was used for statistical tests and visualisation of data. In case of *n* < 5, we performed a Kruskal-Wallis ANOVA followed by Dunn’s post hoc test. For *n* = 5 or more, we tested for normal distribution (ND) and used a one-way ANOVA followed by Tukey’s post hoc test in the case of ND. To test for the interaction of time and treatment, we performed a regular two-way ANOVA followed by Bonferroni’s post hoc test. We assumed *α* = 0.05*.* Statistical tests performed and test results are mentioned in the respective figure captions, the “Results” section and the Supplementary information.

## Results

### Assessment of AgNP and AgNO_3_ behaviour in the exposure medium and exposure conditions in the AIS

TEM imaging of the PVP-AgNP stock dispersion showed small agglomerates and individual particles with a large fraction of 2 nm particles (very low in contrast, Fig. S2). After 10 min, PVP-AgNP dispersions in exposure medium contained larger agglomerates but also individual particles. The hydrodynamic diameter of the stock dispersion, determined by NTA, was 20 ± 13 nm. In exposure medium (5 mg/L AgNP), mean hydrodynamic diameter (NTA) was around 117 ± 7 nm after 3 h and steadily increased within 3 weeks (504 h) to about 256 ± 15 nm, whereas the mode was 117 ± 4 nm after 3 h and decreased to 60 ± 18 nm (Fig. S3, B). DLS measurements also indicated an increase in average size over time (3 h 148 ± 30 nm, 168 h 438 ± 85 nm, Fig. S3, A); however, the PDI increased to >0.5 between 1 and 3 weeks (Fig. S3, A) so that the data for 504 h cannot be used. Mean electrophoretic mobility remained constant around −1.4 μm cm/Vs (Fig. S3, C). Mean total Ag decreased from 4.6 mg/L (90 % nominal Ag concentration) at 3 h to 1.78 mg/L at 24 h (38 % nominal Ag concentration) and remained between 0.64 and 0.68 mg/L till the end of the experiment (504 h, Fig. S3, D). Mean dissolved Ag increased from 0.1 mg/L (2.2 % of total Ag) at 3 h to 0.24 mg/L (13 % of total Ag) at 24 h and remained between 0.31 and 0.2 mg/L at 168 and 504 h (48.5 and 30 % of total Ag, Fig. S3, D). UV-VIS spectra of 5 mg/L PVP-AgNP dispersions showed a SPR peak around 400 nm. The peak intensity decreased between 3 h and 3 weeks to about 40 % (Fig. S4, A). None of the dispersions containing AgNO_3_ (5 mg/L and 20 μg/L) or 20 μg/L PVP-AgNP showed a clear SPR peak above background (Fig. S4, B–E).

Total Ag and DGT-associated Ag in Gauernitzbach and both control AIS were below LOQ. Total Ag was also below LOQ in all AIS before spiking with Ag. In the AgNO_3_-treated AIS, initial concentrations of total Ag were found to be 76 % (Ag2) and 86–95 % (Ag20) of the nominal concentrations on d_0_ (Table 2). Measured total Ag in the AgNP-treated AIS amounted to 68–72 % (NP2) and 81–91 % (NP20) of the nominal concentrations. The fraction of dissolved Ag on d_0_ was between 80 and 92 % in the AgNO_3_-treated AIS and between 32 and 43 % in the AgNP-treated AIS (Table 3). Total Ag rapidly decreased within the first 4 days of the experiment in all treatments. On d_11_, three of four samples from Ag2 and NP2 did not contain Ag above LOQ; the fourth sample (NP2) contained less than 10 % of the nominal concentration. Similarly, the dissolved fraction decreased continuously in the AgNO_3_-spiked AIS down to around 24 % (Ag20) and 0–8 % (Ag2), while the dissolved fraction increased in NP-treated AIS with a maximum on d_11_. The concentration of DGT-associated Ag was in the range of dissolved Ag between d_11_ and d_18_ in all AgNO_3_- and AgNP-treated AIS (Table 2).

**Table 2:** Range of total and dissolved Ag in the AIS measured on d_0_, d_4_, d_11_ and d_18_ and Ag associated with DGT samplers deployed from d_11_ to d_18_ (measured concentration (MC), LOQ 0.01 μg/L) and in percent of the nominal concentration (% NC) relative to the Ag concentration measured in the AgNP and AgNO_3_ stocks. Each AIS was sampled twice at each time point

		Ag2 (2 μg/L)		Ag20 (20 μg/L)		NP2 (μg/L)		NP20 (μg/L)	
		MC [μg/L]	% NC	MC [μg/L]	% NC	MC [μg/L]	% NC	MC [μg/L]	% NC
Total Ag	d_0_	1.37–1.37	75.92–75.92	15.52–17.07	86.22–94.83	1.09–1.16	68.03–72.41	12.94–14.59	80.90–91.21
	d_4_	0.29–0.64	16.10–35.71	1.15–1.44	6.36–7.99	0.28–0.31	17.53–19.34	2.28–2.28	14.27–14.27
	d_11_	<LOD	<LOD	0.48–0.63	2.68–3.50	<LOD–0.15	<LOD–9.41	0.84–0.90	5.24–5.60
	d_18_	0.12–0.12	6.75–6.75	0.26–1.42	7.44–7.86	0.09–0.10	5.84–6.16	0.42–0.45	2.63–2.82
Dissolved Ag	d_0_	1.13–1.26	63.01–69.76	13.7–13.72	76.13–76.24	0.38–0.47	23.83–29.48	4.16–4.86	26.03–30.36
	d_4_	0.17–0.17	9.25–9.25	0.61–0.69	3.42–3.86	0.07–0.14	4.08–8.68	1.47–1.57	9.22–9.79
	d_11_	<LOD	<LOD	0.21–0.29	1.16–1.59	<LOD–0.07	<LOD–4.47	0.82–0.83	5.11–5.20
	d_18_	<LOD–0.01	<LOD–0.56	0.14–0.35	1.76–4.4	<LOD–0.08	<LOD–5.03	0.38–0.39	2.40–2.42
DGT-associated Ag (8 days)	d_11–18_	0.06–0.06	3.17–3.18	0.16–0.17	2.01–2.14	0.04–0.05	2.25–2.94	0.43–0.47	2.68–2.91

**Table 3:** Range of the fraction of dissolved Ag in percent relative to total Ag detected in the AIS. Each AIS was sampled twice at each time point

	Ag2	Ag20	NP2	NP20
d_0_	82.48–91.97	80.37–88.27	32.76–43.12	32.15–33.31
d_4_	26.56–58.62	42.36–60.00	25.00–45.16	64.47–68.86
d_11_	<LOD	43.75–46.03	<LOD	92.22–97.62
d_18_	<LOD–8.33	23.65–24.65	<LOD–88.89	84.44–92.86

**Fig. 1:**
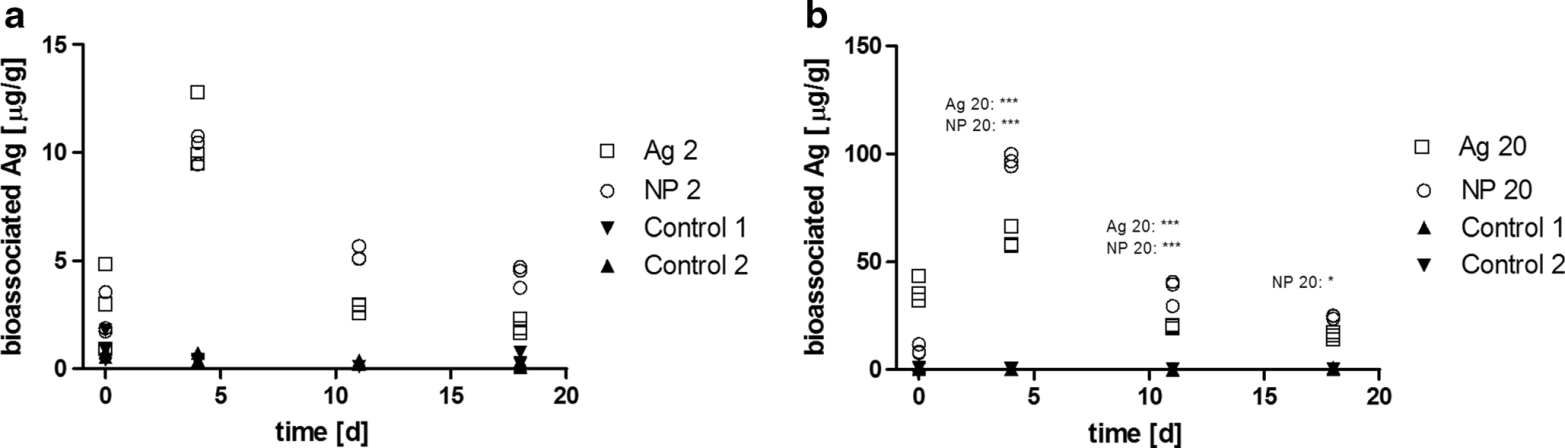
Silver associated with periphyton per tile, based on biomass as dry weight. **a** Control AIS and AIS exposed to 2 μg/L AgNP or AgNO_3_; **b** control AIS and AIS exposed to 20 μg/L AgNP or AgNO_3_. *** and *: significantly different from control 1 and control 2 (*p*_d4_ < 0.0001, *p*_d11_ < 0.0001, *p*_d18_ = 0.0091; *F*_d4_ = 29.12, *F*_d11_ = 99.89, *F*_d18_ = 4.336; one-way ANOVA, Tukey’s post hoc test)

The amount of bioassociated Ag reached a maximum within the first 4 days in all Ag-treated AIS and was proportional to the exposure concentrations (i.e. about one order of magnitude higher in the AIS exposed to a nominal concentration of 20 μg/L Ag than in the AIS exposed to 2 μg/L; Fig. 1, Table S4). Bioassociation of Ag in samples treated with Ag20 was three to four times higher than in those treated with NP20 after around 10 min. The increase of Ag as compared to the controls was significant for Ag20 and NP20 (Fig. 1b), and the trend observed for Ag2 and NP2 was the same but not significant according to two-way ANOVA (Fig. 1a). When expressed relative to the total amount of Ag measured in the exposure medium, the bioassociated Ag seemed to reach an equilibrium within the first 4 days in the AIS Ag20, NP2 and NP20 (Table S4). It decreased in the presence of Ag2. The ratio of bioassociated and total measured Ag was in the same range irrespective of the Ag species and exposure concentration (Table 4).

**Table 4:** Total bioassociated Ag in percent of total Ag measured in the water phase over time in the AIS containing nominal concentrations of 2 or 20 μg/L AgNO_3_ (Ag2, Ag20) or PVP-AgNP (NP2, NP20)

	Ag2	Ag20	NP2	NP20
0 day	0.2	0.15	0.1	0.07
4 days	3	2	2	4
11 days	1	3	2	4
18 days	0.5	2	2	3

### Dynamics of community composition and biomass in control communities

**Fig. 2:**
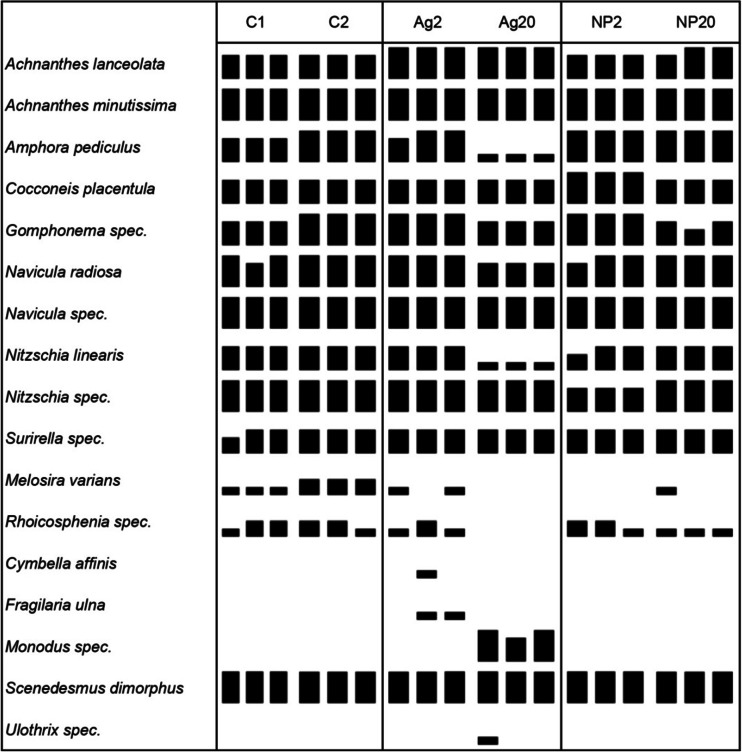
Semi-quantitative species and genera abundance of diatoms and green algae in the six AIS on d_18_ of the experiment. The abundance groups 5 to 1 (5, : >30 %; 4, : 30–10 %; 3, : 10–3 %; 2, : 3–1 %; 1, : <1 %) are depicted as *large* to *small rectangles* for easier interpretation. The groups in numbers are presented in Table S7

At the start of the experiment (d_0_), the abundance of diatoms and green algae was similar in all AIS and all sections of each AIS (Table S5). Within 4 days, the diatoms *Achnanthes lanceolata* ([Brébisson ex Kützing] Grunow in Van Heurck 1880), *Achnanthes minutissima* (Kützing 1833, basionym for *Achnanthidium minutissimum* [Kützing] Czarnecki), *Navicula* sp./*N. radiosa* (Kützing 1844) and *N. linearis* (W. Smith 1853) had increased in abundance more strongly than *A. pediculus* ([Kützing] Grunow ex A. Schmidt 1875), *Cocconeis placentula* (Ehrenberg, 1838), *Gomphonema* sp. (Ehrenberg, 1832) and *Surirella* sp. (Turpin, 1828) (Table S6). After 18 days, *A. minutissima* was the dominant diatom species, followed by *Navicula* sp. and *Nitzschia* sp. (Fig. 2, Table S7). *Melosira varians* and *Rhoicosphenia* sp. (Grunow, 1860) had established themselves in the communities between d_4_ and d_18_, with *M. varians* being slightly more abundant in control 2 than in control 1.

Green algae were less diverse than diatoms. On d_0_, we identified *Scenedesmus dimorphus* ([Turpin] Kützing 1834) and *Monodus* sp. (Chodat, 1913). After 4 days, *Ulothrix* sp. (Kützing 1833) was found in all samples but disappeared again before d_18_. *S. dimorphus* remained the single green algae species at the end of the experiment.

Cyanobacteria were only found sporadically in a few samples and are not listed. Initial POC was not significantly different between the six AIS (median POC 0.006 mg/cm^2^) and increased in the control AIS between d_0_ and d_18_ to a median of 0.118 mg/cm^2^. Dry weight was in the range of 0.2–0.24 mg/cm^2^ on d_18_ in both control AIS.

### Effects of PVP-AgNP and AgNO_3_ on community composition, biomass, EPS and 3D structure of periphyton

On d_4_, diatoms in Ag20-exposed communities showed a reduced abundance compared to the controls and the other treatments. *N. linearis* and *A. pediculus* were not found in any of the Ag20-exposed samples, and *A. lanceolata* and all *Navicula* species except *N. radiosa* were not identified in the sample taken from section B. All green algae communities consisted of *S. dimorphus*, *Monodus* sp. and *Ulothrix* sp. (Table S6). *S. dimorphus* was less abundant in Ag2-, Ag20- and NP20-treated samples, while *Ulothrix* sp. was less abundant in all treated AIS.

After 18 days, *A. minutissima* dominated all diatom communities. *Rhoicosphenia* sp. had established itself in all samples except in the Ag20-treated communities, and *A. pediculus* and *N. linearis* were less abundant in the Ag20 samples than in all the other samples (Fig. 2, Table S7). *M. varians* was found in all controls but only 3 of 12 in Ag- or NP-treated samples. Apart from these differences, the diatom communities exposed to Ag20 seemed to have recovered to a certain extent when compared to the controls. *S. dimorphus* had suppressed all other identifiable green algae except in Ag20-exposed periphyton where *Monodus* sp. remained. *Ulothrix* sp. was identified in one of three samples.

**Fig. 3:**
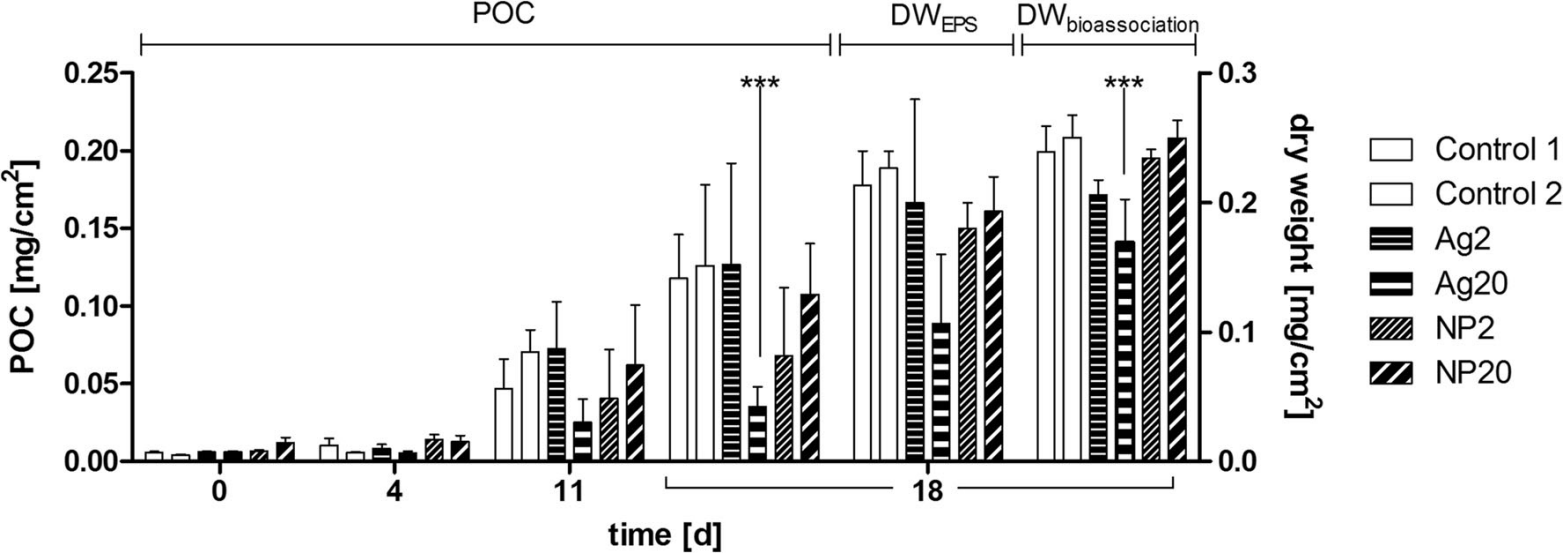
Median values of POC (*n* = 6) and dry weight (*n* = 3) of periphyton [mg/cm^2^] with *bars* indicating the range. ***p < 0.001, significantly different from control 1 and control 2 (two-way ANOVA, Bonferroni’s post-test, Table S3)

**Fig. 4:**
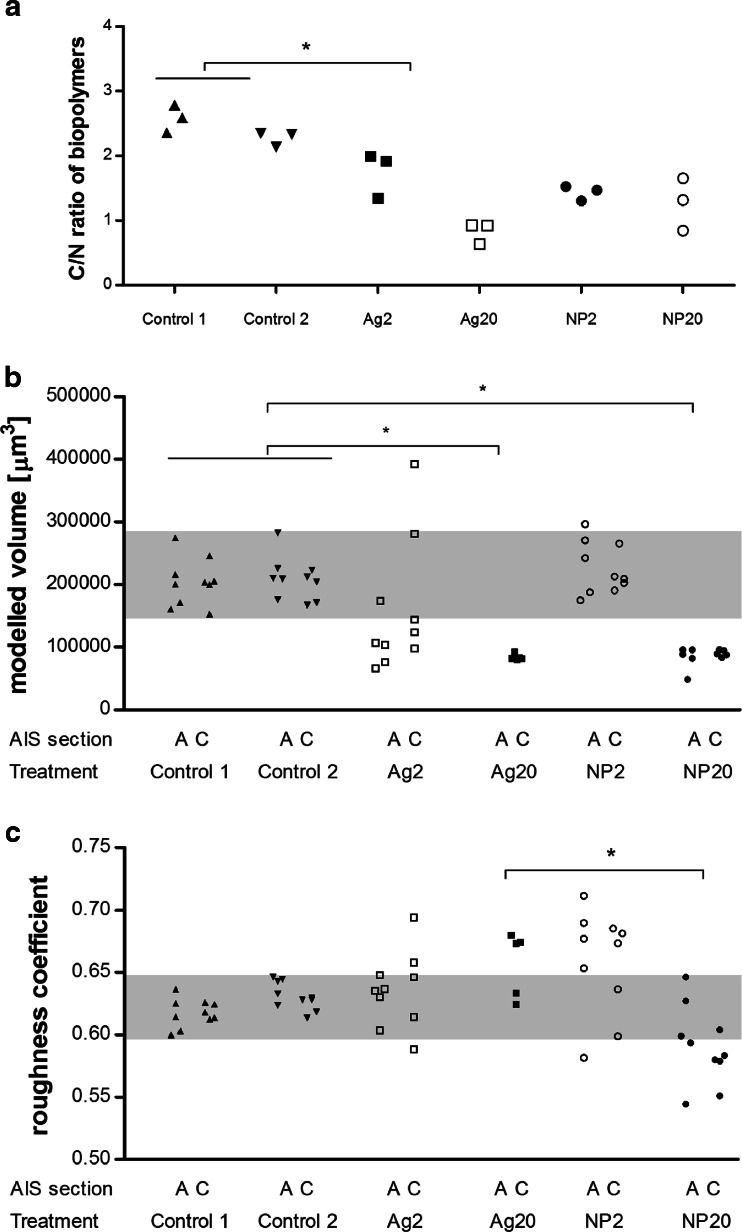
**a** Ratio of organic carbon to organic nitrogen (C/N) in the biopolymer fraction of EPS extracts on d_18_. C/N in Ag20 samples was significantly different from control 1 and control 2 (*p* = 0.014, Kruskal-Wallis, Dunn’s post hoc test). **b** Total volume of chlorophyll-positive, HPA-lectin-positive and reflecting structures modelled based on CLSM data (d_18_). *Each data point* corresponds to one z-stack, five stacks were acquired per patch/tile and two tiles were measured per treatment/AIS except for Ag20 as the biomass was too low on the second randomly sampled tile. *p < 0.0001 (Kruskal-Wallis, Dunn’s post hoc test). **c** Roughness coefficient based on the modelled volume of chlorophyll-positive, HPA-lectin-positive and reflecting structures (d_18_). *Each data point* corresponds to one z-stack, five stacks were acquired per patch/tile and two tiles were sampled per treatment/AIS except for Ag20 as the biomass was too low on the second randomly sampled tile. *p = 0.0068 (Kruskal-Wallis, Dunn’s post hoc test)

The median values of dry weight of the samples for EPS extraction on d_18_ were not significantly different between controls and treatments (Kruskal-Wallis, Dunn’s post hoc test; Fig. 3). Median POC of Ag20-treated biofilms and median dry weight of samples for Ag bioassociation were significantly lower than those of control samples on d_18_ (Fig. 3). All other treatments did not induce significant changes. These differences correspond to the visible heterogeneity in tile coverage in the AIS (see Fig. S10). The differences between POC and DW were not statistically significant (Kruskal-Wallis, Dunn’s post hoc test) (Fig. 4).

The median amount of EPS extracted per dry weight was not significantly different between the controls and treated AIS (Kruskal-Wallis, Dunn’s post hoc test; Fig. S5). Treatments did also not have a significant effect on the fraction of biopolymers, building blocks of HS, low molecular weight (LMW) acids and amphiphilics/neutrals (Fig. S6), and there was no interaction between treatment and EPS components (two-way ANOVA, Bonferroni’s post-test, Table S8). Within the groups, biopolymers and building blocks of HS were always significantly different from LMW acids but only different from amphiphilics/neutrals in samples treated with Ag20 (*p* < 0.001, *t*_polymers_ = 5.802, *t*_building blocks_ = 4.094). No concentration-response relationship of the amount of EPS components versus the initial dissolved Ag concentration could be modelled (Fig. S7). For reference, raw data is provided as chromatograms and integrals thereof in the Supplementary information (Fig. S8).

The ratio of organic carbon to organic nitrogen (C/N) in the biopolymer fraction was significantly lower in the samples from Ag20-treated AIS (0.64–0.92) than in the control samples (2.14–2.79) (*p* = 0.014, Kruskal-Wallis, Dunn’s post hoc test; Fig. 4a). The decrease in C/N in the other treatments was not statistically significant.

The 3D structure of periphyton was assessed on d_18_ by CLSM. The modelled volume of chlorophyll-positive, HPA-lectin-positive and light-reflecting structures was significantly decreased in samples from Ag20- and NP20-treated AIS (Fig. 4b). The decrease in modelled volume of Ag2 samples was not significant due to two outliers. Data from NP2 samples was in the same range as the data from control AIS. A surface roughness coefficient was derived based on the same CLSM data. None of the medians of the treated samples was different from the controls (Fig. 4c). Yet, the variability of the roughness coefficients was increased in the treated samples. The roughness coefficients determined for NP20-treated samples were significantly lower than those determined for Ag20-treated samples.

## Discussion

### Characterisation of the control communities

The variation, measured as diatom and green algae abundance, POC and biomass growth on d_0_ and 18, between the two control AIS was low and proved a general validity of the test conditions for the 18-day testing period. All AIS started with similar abundance of diatoms and green algae for all sections in each AIS on d_0_; also POC was not significantly different between the six AIS. The control channels showed exponential growth indicating a healthy community with comparable dry weights on d_18_.

Bioassociated Ag in the control samples was in the range of <LOQ–1 μg Ag/g dry weight, whereas Ag concentration in Gauernitzbach was below LOQ. The amount of bioassociated Ag in the control samples did not increase over time. We conclude that there was no contamination by Ag from the clay tiles used, although Ag in the range of 0.17 to 1.3 μg Ag/L was extractable from the tiles by soaking in 100 mL 1 % HNO_3_ for 12 h. The background of bioassociated Ag was in the same order of magnitude as the few reported field concentrations of Ag in stream water compared to concentrations in benthic biofilms that we could identify. In the Calcasieu River System and the Bayou D’Inde (both in Louisiana, USA), bioassociation amounted to 0.1–6 and 0.25–1 μg Ag/g biomass, respectively, and concentrations in the stream water were below LOQ or not reported (Ramelow et al. 1987, 1992). In the River Danube, 0.65–2.79 μg Ag/g biomass and 0.01 μg/L dissolved Ag in the stream water were reported (Oertel 1991).

### Characterisation of exposure conditions

#### Characterisation of AgNP in exposure medium

AgNP dispersed in the exposure medium had a higher mean hydrodynamic diameter than in the stock dispersion and increased over time indicating agglomeration in the exposure medium. The mode of the hydrodynamic diameter was identical to the mean in stock and exposure media at day 0, indicating that the particle sizes in suspension were normally distributed. With time, the mode decreased in exposure medium indicating that while the media caused slight agglomeration, the majority of particles remained within the original distribution of particle sizes. UV-VIS spectra showed a decrease in the AgNP SPR peak by ~30 % within the first week and by ~50 % within 3 weeks indicating a continuous decrease in overall AgNP concentration (Fig. S4, A). This decrease in AgNP is reflected in the decrease in total Ag found by ICP-MS (Fig. S3, D). PVP-AgNP have been shown to sediment without particle agglomeration due to their high density (10.490 kg/m^3^) (in the presence of 10 mM CaCl_2_, Jang et al. 2014). Furthermore, the sedimentation rate constant of PVP-AgNP follows Stokes’ law (160.8 nm, *k* = 0.008/h, *t*_1/2_ = 88.9 h; 73.5 nm, k = 0.002/h, *t*_1/2_ = 407.7 h) with the particle settling velocity being proportional to the square of the particle diameter and the density difference between particle and liquid. Our data thus reflect the slight agglomeration and faster proportional loss of larger particles and agglomerates over time, probably by NP density-driven sedimentation.

The lack of a distinct SPR peak in AgNO_3_ dispersions indicates that no detectable AgNP formation took place through (photo-)reduction of Ag^+^ under the chosen conditions. We accept the limitations of comparison between the characterisation samples and the exposure setup due to particle behaviour in aquatic test media being concentration dependent (e.g. Piccapietra et al. 2011). It is clear that the biological dynamics in the exposure system are likely to have influenced AgNP behaviour and have done so in a different manner than the dynamics known from in vitro approaches. The effects of e.g. direct NP-organism interactions in the communities which changed in their composition and the release of species-specific EPS are not known, nor can they be followed in in vivo experiments due to the gap between the limits of detection of characterisation techniques (high μg/L to mg/L range for DLS, depending on primary particle size; Bundschuh et al. 2001) and TEM (Matzke et al. 2014), although possibly getting close to the 50–100-μg/L technical limit of NTA in clean media and the sensitivity of the organisms.

#### Dynamics of Ag concentrations in the AIS

In a previous in vitro study performed in 10 mM CaCl_2_, PVP-AgNP have been shown to sediment without particle agglomeration with a sedimentation rate constant *k* = 0.008/h and *t*_1/2_ = 88.9 h (3.7 days) for particles with a primary particle size of 160.8 nm (Jang et al. 2014). The decrease to about 50 % of AgNP within 4 days in the water phase in the presented study (both in AgNP dispersions and in the AIS) is in a similar range and could be due to the same process. The decrease in total Ag in both the AgNP- and AgNO_3_-treated AIS and the opposite trend in the fraction of dissolved Ag in AgNP- (increasing) and AgNO_3_-treated (decreasing) AIS (Table 3) indicate that the dissolved fraction of the silver in the water phase was lost faster than the non-dissolved. So while both total and dissolved Ag were higher in the Ag20 AISs at d_0_ than in the NP20 AIS, then from d_4_ onwards, both total and dissolved Ag were marginally higher in the NP20 AIS (Table 2), as the remaining suspended gaps slowly dissolved. The absence of this pattern in the NP2 treatment may be due to either faster dissolution or faster loss to surfaces of the non-dissolved phase at the lower concentration. The exposure medium used in the presented study was chosen to resemble stream water like the one that the biofilms had been sampled from. Consequently, Ag speciation was not optimised to provide the highest possible concentration of free Ag^+^. Based on the equilibrium speciation model Visual MINTEQ, we expected around 90 % (Ag20) and 40 % (Ag2) of the total Ag being present as Ag^+^ at equilibrium at 15 °C (Tables S9 and S10). However, these predicted values only corresponded to measured concentrations at the start of the exposure in the Ag20 AIS. In all other treatments, measured Ag concentrations were clearly below the expected concentrations. Possible explanations are the binding to surfaces of the AIS system or formation of particulate Ag, or a combination thereof. Based on the in vitro characterisation of AgNO_3_ in exposure medium at higher concentrations (lack of SPR signal in UV-VIS spectra), we do not have any indication of the formation of elemental AgNP. However, as periphytic EPS, humic and fulvic acids have been shown to induce (photo)reduction of Ag^+^ to AgNP in vitro (Adegboyega et al. 2013; Akaighe et al. 2011; Kroll 2014), this process can also not be ruled out. At the same time, PVP may have detached from the NP surface and/or have been metabolised over time exposing more AgNP surface and making it prone to corrosion. As NOM concentrations were not different between the AIS (1.58–1.79 mg/L, Table S1) and the spectra recorded by LC-OCD-OND did not vary (Fig. S9), we conclude that the difference in the fraction of dissolved Ag in AgNO_3_- and AgNP-exposed AIS was not explainable by a change in DOC properties or amounts.

In general, the decrease of total Ag in the AIS corresponds to observations in a recent mesocosm study although the exposure concentrations in these mesocosms were two to three orders of magnitude higher. In the study of Colman et al. (2014), the initial concentrations of 2.5 mg/L Ag (AgNO_3_, PVP and gum arabic-coated AgNP) decreased to below 0.5 mg/L within the first 6 days of the experiment following first-order decay kinetics independent of the Ag species applied. The authors speculated that Cl^−^ released from plants growing in the mesocosms may have precipitated Ag^+^ from the water phase. According to our results, the major predicted species apart from Ag^+^ was AgCl (VMINTEQ, Table S10). At equilibrium, the concentration of Cl^−^ would have had to increase by an order of magnitude (10^−2^ to 10^−1^ g/L) to decrease the fraction of Ag^+^ by an order of magnitude (10^−5^ to 10^−6^ g/L), which was not the case (range of Cl^−^ medians, 24.5–29.7 mg/L, Table S1). There are certain limitations to VMINTEQ that may be the reason for the observed discrepancy: The model can neither take into account the dissolution and settling kinetics of AgNP nor the formation of AgNP from Ag^+^ in the exposure system. Due to continued biological processes in the AIS, the system may also not reach an equilibrium. The temperature-corrected solubility constants *K*_s_ used by VMINTEQ are either adjusted by taking into account the reaction enthalpy or empirical relationships for a small number of species. Thus, the fraction of Ag precipitated as AgCl may have been larger than predicted by the equilibrium model.

The amount of bioassociated Ag was proportional to the order of magnitude of the total measured Ag which indicates that uptake was limited by diffusion across the DBL (Tessier 1994). Ten minutes after the start of exposure, measured bioassociation of Ag was three to four times higher in Ag20- than in NP20-treated samples which corresponds to the ratio of dissolved Ag in the two treatments (2.5–2.9, calculated from Table 2). On d_4_–d_18_, maximum measured amounts of bioassociated Ag in NP20 and NP2 were up to twice as high as those found in Ag20 and Ag2, respectively, whereas the medians were not significantly different. This trend could indicate a slower association with and longer retention of Ag in the biofilms when delivered as AgNP and should be examined in experiments with a higher number of replicates providing a statistical power useful to determine whether the differences are significant.

The decrease in overall biomass concentration of Ag over time is probably due to growth dilution as reported previously for other metals (Hill and Larsen 2005): The concentration of total and dissolved Ag in the water decreased within the first 4 days; thus, the major fraction of Ag must have associated with the biofilms in the first days after spike exposure while it was still available. As biomass steadily increased over time, the ratio of bioassociated Ag to biomass decreased accordingly.

To our knowledge, data on bioassociated Ag in periphyton from controlled studies are not available as of now. Compared to field data, the ratio of bioassociated Ag [μg/g] and Ag in the water phase [μg/L] we observed in the Ag-treated AIS after 18 days was one to two orders of magnitude higher than those observed in River Danube (Oertel 1991). We suggest that this difference may be due to several factors: the Ag speciation in the AIS not having reached equilibrium yet and differences in availability due to different water chemistry, different biomass per area and/or different species composition. Compared to experiments in heterotrophic monospecies biofilms and suspended algal cultures, even the maximal bioassociation observed on d_4_ was at least one order of magnitude lower, but showed the same linear relationship between exposure concentration in the range of micrograms per litre and bioaccumulation (Fabrega et al. 2009; Forsythe et al. 1996; Leclerc and Wilkinson 2013; Piccapietra et al. 2012). In the green alga *Scenedesmus obliquus*, accumulation of ^110m^Ag depended on the Ag concentration and the amount of biomass (Garnier and Baudin 1989). Accumulation of Ag was up to two orders of magnitude higher than from Ag_2_ S_2_O_3_^2−^, which the authors explained by difference in Ag species and exposure protocols.

### Effects of PVP-AgNP and AgNO_3_ to periphyton

#### Diversity and population density of diatoms and green algae

As summarised by Gray (1997) for marine ecosystems, the potential responses of a community to pollution include (1) reduced diversity, (2) increased dominance of opportunistic species and (3) reduced number of individuals. Our data suggest that exposure to Ag20 reduced the diversity and population density of *A. pediculus*, *N. radiosa* and *N. linearis* and eliminated *Rhoicosphenia* sp., while no differences were detected between the control communities and those exposed to Ag2, NP2 and NP20. Diatom diversity and abundance decreased within the first 4 days of exposure to Ag20 compared to all other treatments, but recovered to a certain extent by d_18_. The Ag20 exposure also had a negative impact on *S. dimorphus* abundance on d_4_ which is possibly the reason for *Monodus* sp. still occupying a niche in the presence of Ag20 on d_18_, while it had been displaced in all other AIS.

A decrease in diatom abundance could have been caused by a limitation of Si or decreased the bioavailability of Si. Total measured Si did not differ in the AIS (range of medians 4.4–6.1 mg/L, Table S1), and according to VMINTEQ, dissolved Si should have been the same in all AIS (46.39 %, Table S9). The P/N ratio, which could have been an explanation for the variation in taxa, also showed neither change over time nor varied among the AIS.

The population density of the diatoms *A. pediculus*, *N. linearis* and *Rhoicosphenia* sp. was affected by exposure to Ag20. From our data, we could not infer any preference for motile or sessile diatoms in the Ag20 samples as *N. linearis* and *A. pediculus* are motile, whereas *A. minutissima*, which dominated the diatom community in all AIS, and *Roicosphenia* sp. are sessile. Similar to our observed non-sensitivity of *A. minutissima* to the Ag exposures, it has been described as one of the most metal-tolerant species (Cantonati et al. 2014; Medley and Clements 1998) and was the primary replacement species in benthic biofilms continuously exposed to copper in Convict Creek (Sierra Nevada, USA) (Leland and Carter 1984, 1985). The abundance of *M. varians*, which was clearly affected by exposure to Ag, was also negatively correlated with cadmium concentrations in the Riou-Mort River (Duong et al. 2008).

As exposure to NP20 did not induce the same changes in community composition as Ag20, while bioassociation was similar or even higher in NP20-exposed biofilms, we suggest that the initial concentration of dissolved Ag (~13.7 μg/L) in the Ag20 treatment interfered with the establishment of Ag-sensitive species in the biofilms. Diatom communities started recovering within 18 days in the Ag20 exposure, also indicating that the initial exposure conditions caused a knockdown from which recovery was possible as the dissolved and total Ag concentrations reduced in time. The initial concentration of dissolved Ag in the other treatments (~0.4–4.9 μg/L, see Table 2) did not affect community composition.

#### EPS concentration and composition

EPS have been suggested to play a role in complexing and detoxifying heavy metals (Geesey 1982). Relevant functional groups in EPS would be negatively charged such as carboxylic or sulfhydrylic groups. A general increase in EPS per biomass in the presence of AgNP (unknown concentration) was observed in the marine diatom *Thalassiosira weissflogii* (Miao et al. 2009) and in *E. coli* (Joshi et al. 2012) as well as bacteria in activated sludge (100 μg/L and below) (Zhang et al. 2014). Based on these experiments, a concentration-dependent increase of EPS was expected. In contrast, the median EPS composition was not significantly different between treatments and controls, although three of the AgNO_3_-treated samples had a higher EPS concentration per dry weight than all of the control and AgNP-treated samples. However, 18 days after the addition of Ag, the range of EPS concentration of samples from all treated AIS was higher than EPS from control AIS. The variability in EPS concentration and composition observed may be due to (1) an increase in heterogeneity or patchiness of the biofilms below the resolution chosen here and/or (2) a change in the heterotrophic community. Both responses may have resulted in (a) a local selection for photo- and heterotrophic individuals expressing a higher amount for EPS/EPS components and/or (b) local induction of EPS/EPS component production.

The C/N ratio in the biopolymer fraction was significantly decreased in Ag20-treated samples indicating an increase in high molecular weight proteins. As the concentrations of total and dissolved Ag on d_18_ did not show the same trend, it is unlikely that the differences in C/N ratio were due to detectable depletion of polysaccharides by Ag species. In experiments with the algae species *T. weissflogii*, exposure to Ag (AgNO_3_ and AgNP) correlated with an increase in the amount of polysaccharides in the biopolymer fraction (Miao et al. 2009). The observed decrease in C/N ratio in the biopolymer fraction may thus not be a common response, but the sum of all C/N changes in response to exposure to 20 μg/L AgNO_3_. A recent short-term (2 h exposure) study revealed that citrate-coated AgNP and AgNO_3_ affected the activity of two extracellular enzymes in EPS extracted from periphyton in a concentration-dependent manner (Gil-Allué et al. 2014). In all cases, the effective concentrations of the ionic silver exposures were lower than those of the AgNP exposures when effects were expressed based on dissolved Ag. Based on control experiments, the authors concluded that the reduced enzymatic activity was due to reduced secretion. Follow-up experiments on extracellular enzyme activity and EPS composition are needed to link the observed changes and to test whether the decrease in C/N ratio is a function of the concentration of initially dissolved Ag or whether other factors such as Ag speciation influence the C/N ratio.

#### 3D structure: modelled volume and roughness coefficient

Exposure to Ag20 and NP20 significantly decreased the modelled volume to about 50 % compared to the median of the controls. The variability of the modelled volumes was much lower in Ag20- and NP20-treated samples as compared to the controls. As the biomass of Ag20- and NP20-treated samples did not decrease to the same extent as the biofilm volumes decreased, our data indicate that Ag-exposed biofilms became locally denser than the controls.

The roughness coefficients were significantly lower in NP20-treated samples as opposed to those treated with Ag20. As we did not observe any opposing differences in any of the other endpoints, we suggest that this difference might be specific to the Ag species applied. The range of the roughness coefficients was wider in treated samples compared to those taken from control AIS indicating increased local heterogeneity of the 3D structure in the Ag-exposed communities.

The existence and composition of periphyton influence near-bed hydrology, roughness length and resistance to flow in streams (Nikora et al. 1997, 1998). Additionally, biofilm structure may influence the thickness of the DBL which influences the exchange of dissolved compounds and colloids between the biofilm and the water phase (Jørgensen and Des Marais 1990). The impact of pollutants on periphyton structure in the range of micrometres has not yet been studied to our knowledge.

## Conclusions

Although the bulk of the Ag disappeared from the water phase within the first 4 days of exposure, bioassociated Ag above background levels was still detectable 18 days after spike exposure. The parameters chosen to characterise periphyton revealed the effects of the initial Ag^+^ concentration and the effects specific to the silver species applied. Diatom and green algae communities, biomass and EPS composition were impacted by 20 μg/L AgNO_3_ (nominal concentration) indicating that the effective threshold was between 4.9 μg/L Ag^+^ (20 μg/L AgNP, no effect) and 13.7 μg/L Ag^+^ (20 μg/L AgNO_3_ significant changes) in this setup. While diatom abundance and diversity was reduced by 20 μg/L AgNO_3_, it kept *S. dimorphus* from dominating the green algae community, indicating that exposure to silver may have opposite effects on the biodiversity of these communities. The decreased C/N ratio of extracellular biopolymers indicates a decreased ratio of polysaccharides to proteins which is potentially relevant to the interaction of periphyton with particles/colloids and dissolved substances. As opposed to the other endpoints, the 3D structure of periphyton pointed to the effects specific to the Ag species applied. Both 20 μg/L AgNO_3_ and AgNP significantly decreased biofilm volume to about 50 % compared to the controls; however, the decrease of the biomass was much lower in 20 μg/L AgNP-treated samples than in the 20-μg/L AgNO_3_ samples. Together, these observations indicate a compaction of AgNP-exposed biofilms, which may be related to the unchanged C/N ratio of their extracellular biopolymers. The decrease in roughness coefficient was higher in 20 μg/L AgNP-treated samples, which may affect the thickness of the diffusive boundary layer. Based on these results, future studies should be aimed at understanding whether changes in EPS and 3D structure follow a concentration-response relationship and at what level these changes affect the functions of EPS and near-bed hydrology.

Our results show that the interpretation of long-term effects of different Ag species on periphyton will depend on the endpoints chosen, which is potentially valid when comparing other metal nanoparticle-metal ion pairs. The more traditional endpoints biomass and diversity indicated a concentration-dependent effect of silver ions. Newly introduced parameters that measure both the extracellular chemical environment and 3D structure of periphyton indicated both concentration-dependent effects of silver ions and effects related to the silver species applied. We suggest that tools allowing the combination of biofilm structure with function under stress as presented here should be complemented with other functional endpoints like extracellular enzyme activity, photosynthetic yield, oxygen production and potential unknown descriptors in a toolbox to fully understand the potential impact of pollution-induced changes in periphyton communities and structures upon ecosystem functions.

## Electronic supplementary material

ESM 1 (DOCX 2848 kb)

## References

[CR1] Adegboyega NF, Sharma VK, Siskova K, Zbořil R, Sohn M, Schultz BJ, Banerjee S (2013). Interactions of aqueous Ag+ with fulvic acids: mechanisms of silver nanoparticle formation and investigation of stability. Environ Sci Technol.

[CR2] Akaighe N, MacCuspie RI, Navarro DA, Aga DS, Banerjee S, Sohn M, Sharma VK (2011). Humic acid-induced silver nanoparticle formation under environmentally relevant conditions. Environ Sci Technol.

[CR3] Azim ME, Verdegem MCJ, van Dam AA, Beveridge MCM (2006). Periphyton: ecology, exploitation and management.

[CR4] Bellinger BJ, Gretz MR, Domozych DS, Kiemle SN, Hagerthey SE (2010). Composition of extracellular polymeric substances from periphyton assemblages in the Florida everglades. J Phycol.

[CR5] Bondarenko O, Juganson K, Ivask A, Kasemets K, Mortimer M, Kahru A (2013). Toxicity of Ag, CuO and ZnO nanoparticles to selected environmentally relevant test organisms and mammalian cells in vitro: a critical review. Arch Toxicol.

[CR6] Borgmann U (1996). Systematic analysis of aqueous ion requirements of Hyalella azteca: a standard artificial medium including the essential bromide ion. Arch Environ Contam Toxicol.

[CR7] Bundschuh T, Knopp R, Kim JI (2001). Laser-induced breakdown detection (LIBD) of aquatic colloids with different laser systems. Colloids Surf A: Physicochem Eng Asp.

[CR8] Cantonati M, Cantonati M, Cantonati M (2014). Achnanthidium minutissimum (Bacillariophyta) valve deformities as indicators of metal enrichment in diverse widely-distributed freshwater habitats. Sci Total Environ.

[CR9] Colman BP (2014). Emerging contaminant or an old toxin in disguise? Silver nanoparticle impacts on ecosystems. Environ Sci Technol.

[CR10] Dade WB (1993). Near-bed turbulence and hydrodynamic control of diffusional mass transfer at the sea floor. Limnol Oceanogr.

[CR11] Davlson W, Zhang H (1994). In situ speciation measurements of trace components in natural waters using thin-film gels. Nature.

[CR12] Dodds WK, Biggs BJF (2002). Water velocity attenuation by stream periphyton and macrophytes in relation to growth form and architecture. J N Am Benthol Society.

[CR13] DREWAG (2013) WW Coschütz Reinwasser – Statistische Auswertung der Analysendaten 2013. https://www.drewag.de/…/de/reinwasser_coschuetz.pdf. Accessed 21 January 2015

[CR14] Duong TT, Morin S, Herlory O, Feurtet-Mazel A, Coste M, Boudou A (2008). Seasonal effects of cadmium accumulation in periphytic diatom communities of freshwater biofilms. Aquat Toxicol.

[CR15] Evanoff DD, Chumanov G (2005). Synthesis and optical properties of silver nanoparticles and arrays. Chemphyschem.

[CR16] Fabrega J, Renshaw JC, Lead JR (2009). Interactions of silver nanoparticles with Pseudomonas putida biofilms. Environ Sci Technol.

[CR17] Flemming HC, Wingender J (2010). The biofilm matrix. Nat Rev Microbiol.

[CR18] Forsythe BI, La Point T, Cobb G, Klaine S Silver in an experimental freshwater ecosystem. In: 4th Argentum international conference on the transport, fate, and effects of silver in the environment, Madison, WI, USA, 1996. pp 185–189

[CR19] Garnier J, Baudin JP (1989). Accumulation and depuration of ^110m^Ag by a planktonic alga, Scenedesmus obliquus. Water, Air, Soil Pollut.

[CR20] Geesey G (1982). Microbial exopolymers: ecological and economic considerations. ASM Am Soc Microbiol News.

[CR21] Gil-Allué C, Schirmer K, Tlili A, Gessner MO, Behra R (2014). Silver nanoparticle effects on stream periphyton during short-term exposures. Environ Sci Technol.

[CR22] Gray J (1997). Marine biodiversity: patterns, threats and conservation needs. Biodivers Conserv.

[CR23] Hamm A (1991). Studie über die Wirkungen und Qualitätsziele von Nährstoffen in Fließgewässern vol 1.

[CR24] Harrison JJ, Ceri H, Yerly J, Stremick CA, Hu Y, Martinuzzi R, Turner RJ (2006). The use of microscopy and three-dimensional visualization to evaluate the structure of microbial biofilms cultivated in the Calgary biofilm device. Biol Proc Online.

[CR25] Hill WR, Larsen IL (2005). Growth dilution of metals in microalgal biofilms. Environ Sci Technol.

[CR26] Hürlimann J. NP (2007) Methoden zur Untersuchung und Beurteilung der Fliessgewässer. Kieselalgen Stufe F (flächendeckend). vol Umwelt-Vollzug Nr. 0740. Bundesamt für Umwelt, Bern

[CR27] Hustedt F (1976) Bacillariophyta (Diatomeae). 2. Aufl., Repr. [d. Ausg.] Jena, Fischer, 1930 edn. Koeltz Königstein

[CR28] Ivask A (2014). Size-dependent toxicity of silver nanoparticles to bacteria, yeast, algae, crustaceans and mammalian cells in vitro. PLoS One.

[CR29] Jang MH, Bae SJ, Lee SK, Lee YJ, Hwang YS (2014). Effect of material properties on stability of silver nanoparticles in water. J Nanosci Nanotechnol.

[CR30] Jørgensen BB, Des Marais DJ (1990). The diffusive boundary layer of sediments: oxygen microgradients over a microbial mat. Limnol Oceanogr.

[CR31] Joshi N, Ngwenya BT, French CE (2012). Enhanced resistance to nanoparticle toxicity is conferred by overproduction of extracellular polymeric substances. J Hazard Mater.

[CR32] Jungmann D, Brust K, Licht O, Mählmann J, Schmidt J, Nagel R (2001). Artificial indoor streams as a method to investigate the impact of chemicals on lotic communities. Environ Sci Pollut Res.

[CR33] Kang F, Alvarez PJ, Zhu D (2013). Microbial extracellular polymeric substances reduce Ag+ to silver nanoparticles and antagonize bactericidal activity. Environ Sci Technol.

[CR34] Kroll A (2014). Extracellular polymeric substances (EPS) of freshwater biofilms stabilize and modify CeO2 and Ag nanoparticles. PLoS One.

[CR35] Labiod C, Godillot R, Caussade B (2007). The relationship between stream periphyton dynamics and near-bed turbulence in rough open-channel flow. Ecol Model.

[CR36] Lamberti GA, Stevenson RJ, Bothwell ML, Lowe RL (1996). The role of periphyton in benthic food webs. Algal ecology: freshwater benthic ecosystems.

[CR37] Larned ST (2010). A prospectus for periphyton: recent and future ecological research. J N Am Benthol Soc.

[CR38] Leclerc S, Wilkinson KJ (2013). Bioaccumulation of nanosilver by Chlamydomonas reinhardtii—nanoparticle or the free ion?. Environ Sci Technol.

[CR39] Leland HV, Carter JL (1984). Effects of copper on species composition of periphyton in a Sierra Nevada, California, stream. Freshw Biol.

[CR40] Leland HV, Carter JL (1985). Effects of copper on production of periphyton, nitrogen fixation and processing of leaf litter in a Sierra Nevada, California, stream. Freshw Biol.

[CR41] Licht O, Jungmann D, Ludwichowski KU, Nagel R (2004). Long-term effects of fenoxycarb on two mayfly species in artificial indoor streams. Ecotox Environ Safe.

[CR42] Matzke M, Jurkschat K, Backhaus T (2014). Toxicity of differently sized and coated silver nanoparticles to the bacterium Pseudomonas putida: risks for the aquatic environment?. Ecotoxicology.

[CR43] Medley CN, Clements WH (1998). Responses of diatom communities to heavy metals in streams: the influence of longitudinal variation. Ecol Appl.

[CR44] Miao AJ, Schwehr KA, Xu C, Zhang SJ, Luo Z, Quigg A, Santschi PH (2009). The algal toxicity of silver engineered nanoparticles and detoxification by exopolymeric substances. Environ Pollut.

[CR45] Murga R, Stewart PS, Daly D (1995). Quantitative analysis of biofilm thickness variability. Biotechnol Bioeng.

[CR46] Navarro E (2008). Toxicity of silver nanoparticles to Chlamydomonas reinhardtii. Environ Sci Technol.

[CR47] Neu TR, Lawrence JR, Ron JD (1999). Lectin-binding analysis in biofilm systems. Methods in enzymology.

[CR48] Nikora VI, Goring DG, Biggs BJF (1997). On stream periphyton-turbulence interactions New Zealand. J Mar Freshw Res.

[CR49] Nikora VI, Goring DG, Biggs BJF (1998). A simple model of stream periphyton-flow interactions. Oikos.

[CR50] Nowicki B (1985). Multiparameter representation of surface roughness. Wear.

[CR51] Oertel N (1991). Heavy-metal accumulation in Cladophora glomerata (L.) Kutz in the River Danube. Ambio.

[CR52] Pascher A, Büdel, B., Ettl, H. (1987-1997). Fischer, Jena, Stuttgart

[CR53] Petering HG (1976). Pharmacology and toxicology of heavy metals: silver. Pharmacology & Therapeutics Part A. Chem, Toxicol Metab Inhib.

[CR54] Piccapietra F, Sigg L, Behra R (2011). Colloidal stability of carbonate-coated silver nanoparticles in synthetic and natural freshwater. Environ Sci Technol.

[CR55] Piccapietra F, Allué CG, Sigg L, Behra R (2012). Intracellular silver accumulation in Chlamydomonas reinhardtii upon exposure to carbonate coated silver nanoparticles and silver nitrate. Environ Sci Technol.

[CR56] Pillai S, Behra R, Nestler H, Suter MJ-F, Sigg L, Schirmer K (2014). Linking toxicity and adaptive responses across the transcriptome, proteome, and phenotype of Chlamydomonas reinhardtii exposed to silver. Proc Natl Acad Sci U S A.

[CR57] Ramelow GJ, Maples RS, Thompson RL, Mueller CS, Webre C, Beck JN (1987). Periphyton as monitors for heavy metal pollution in the Calcasieu River estuary. Environ Pollut.

[CR58] Ramelow GJ, Biven SL, Zhang Y, Beck JN, Young JC, Callahan JD, Marcon MF (1992). The identification of point sources of heavy metals in industrially impacted waterway by periphyton and surface sediment monitoring. Water, Air, Soil Pollut.

[CR59] Romano PR (2011). Development of recombinant Aleuria aurantia lectins with altered binding specificities to fucosylated glycans. Biochem Biophys Res Commun.

[CR60] Rybicki M, Winkelmann C, Hellmann C, Bartels P, Jungmann D (2012). Herbicide indirectly reduces physiological condition of a benthic grazer. Aquat Biol.

[CR61] Salant NL (2011). ‘Sticky business’: the influence of streambed periphyton on particle deposition and infiltration. Geomorphology.

[CR62] Staudt C, Horn H, Hempel DC, Neu TR (2004). Volumetric measurements of bacterial cells and extracellular polymeric substance glycoconjugates in biofilms. Biotechnol Bioeng.

[CR63] Stewart T, Traber J, Kroll A, Behra R, Sigg L (2013). Characterization of extracellular polymeric substances (EPS) from periphyton using liquid chromatography-organic carbon detection–organic nitrogen detection (LC-OCD-OND). Environ Sci Pollut Res.

[CR64] Tessier A (1994) Uptake of trace metals by aquatic organisms. Chemical and Biological Regulation of Aquatic Systems

[CR65] Tourney J, Ngwenya BT (2014). The role of bacterial extracellular polymeric substances in geomicrobiology. Chem Geol.

[CR66] Wagner M, Ivleva NP, Haisch C, Niessner R, Horn H (2009). Combined use of confocal laser scanning microscopy (CLSM) and Raman microscopy (RM): investigations on EPS-matrix. Water Res.

[CR67] Webb JS (2003). Cell death in Pseudomonas aeruginosa biofilm development. J Bacteriol.

[CR68] West S, Horn H, Hijnen WAM, Castillo C, Wagner M (2014). Confocal laser scanning microscopy as a tool to validate the efficiency of membrane cleaning procedures to remove biofilms. Sep Purif Technol.

[CR69] Winkelmann C, Petzoldt T, Koop JHE, Matthaei CD, Benndorf J (2008). Benthivorous fish reduce stream invertebrate drift in a large-scale field experiment. Aquatic Ecol.

[CR70] Zhang C, Liang Z, Hu Z (2014). Bacterial response to a continuous long-term exposure of silver nanoparticles at sub-ppm silver concentrations in a membrane bioreactor activated sludge system. Water Res.

