# Supplementary material for: Mixed messages from benthic microbial communities exposed to nanoparticulate and ionic silver: 3D structure picks up nano-specific effects, while EPS and traditional endpoints indicate a concentration-dependent impact of silver ions
Source: Environ Sci Pollut Res Int. 2015 Jun 28;23:4218–34. doi: 10.1007/s11356-015-4887-7 (PMC4766215; doi:10.1007/s11356-015-4887-7)
Supplement: Supplementary file 1 — (DOCX 2848 kb) [file 11356_2015_4887_MOESM1_ESM.docx]

**Supplementary information**

**Title**

Mixed messages from benthic microbial communities exposed to nanoparticulate and ionic silver: 3D structure picks up nano-specific effects while EPS and traditional endpoints indicate a concentration-dependent impact of silver ions

**Authors**

Alexandra Kroll^1$*^, Marianne Matzke^$2^, Marcus Rybicki^3^, Patrick Obert-Rauser^3^, Corinna Burkart^3^, Kerstin Jurkschat^4^, Rudo Verweij^5^, Linn Sgier^1^, Dirk Jungmann^3^, Thomas Backhaus^6^, Claus Svendsen^2^

^1^ Eawag, Swiss Federal Institute of Aquatic Science and Technology, 8600 Dübendorf, Switzerland

^2^ NERC Centre for Ecology and Hydrology, Acremann Section, Ecotoxicology, Maclean Building, Benson Lane, Crowmarsh Gifford, OX10 8BB, United Kingdom

^3^ Technical University of Dresden, Faculty Environmental Sciences, Institute for Hydrobiology, Zellescher Weg 40, 01217 Dresden, Germany

^4^ Department of Materials, Oxford University Begbroke Science Park, Begbroke Hill, Yarnton, Oxford OX5 1PF, United Kingdom

^5^ Vrije Universiteit Amsterdam, Faculty of Earth and Life Sciences, Institute of Ecological Science, Department of Animal Ecology, De Boelelaan 1085, 1081 HV Amsterdam, The Netherlands

^6^ University of Gothenburg, Department of Biological and Environmental Sciences, Carl Skottsbergs Gata 22 B, 40530 Göteborg/Sweden

^$^ these authors contributed equally

* alexandra.kroll@eawag.ch

***Figures, Tables, and Text are presented in the order of appearance in the main text***

**1. Supplementary Figures**


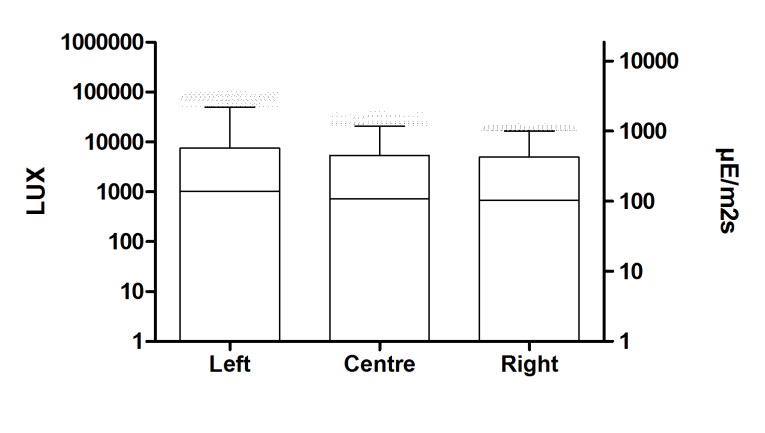

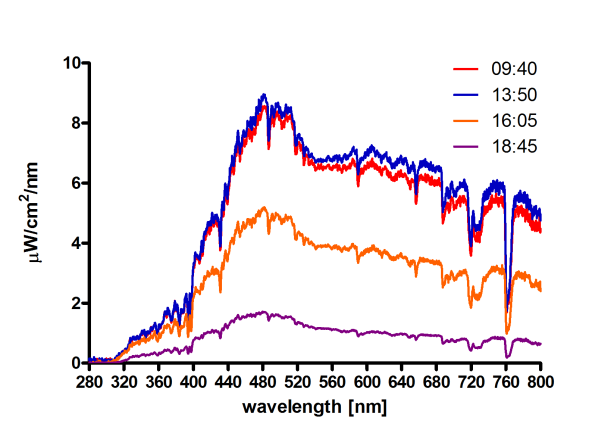


Figure S1: Light conditions in the greenhouse. Left panel: Irradiation from sunrise to sunset at three positions plotted in LUX and µE/m^2^s, box-plots with 5-95% whiskers. Right panel: Representative spectra of incident light at the centre of the greenhouse over the course of a day.


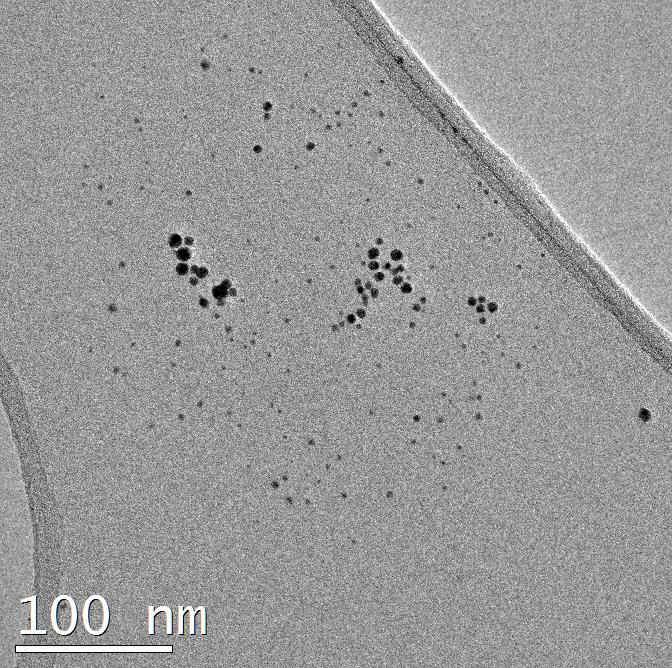

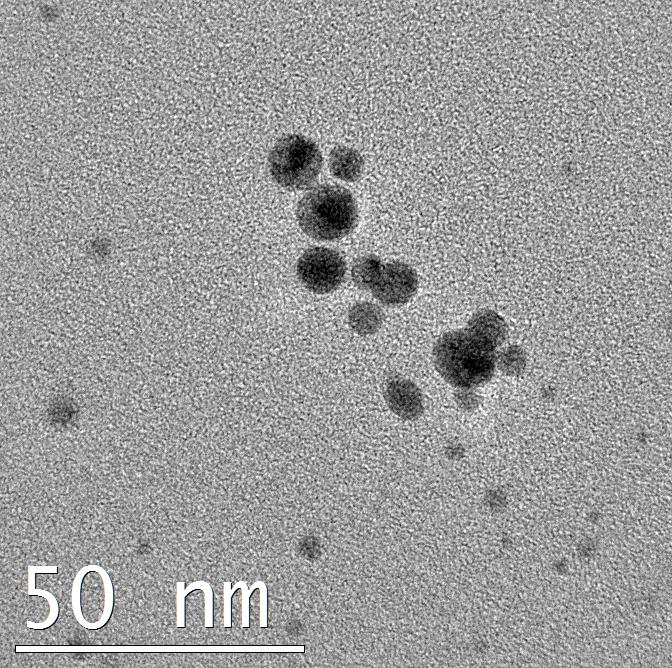


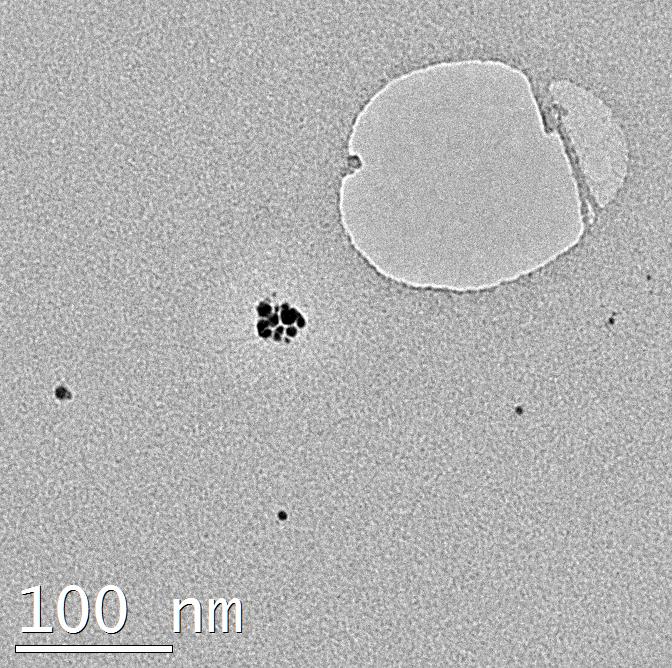

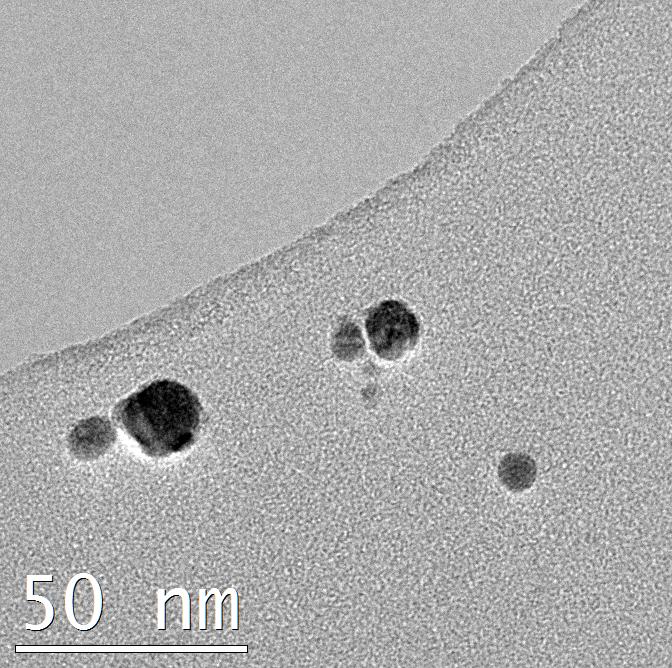


Figure S2: TEM images of PVP-AgNP. Upper row: stock dispersion (particles are mostly spherical in shape), lower row: 1 mg/L in the exposure medium after 1 h (particles have reacted with the exposure medium and show irregular shapes).


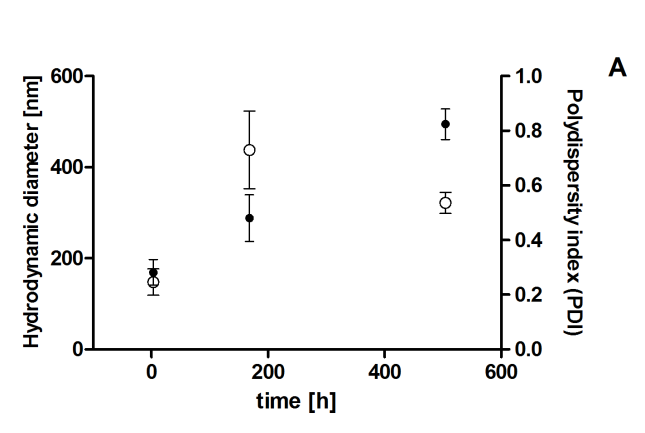

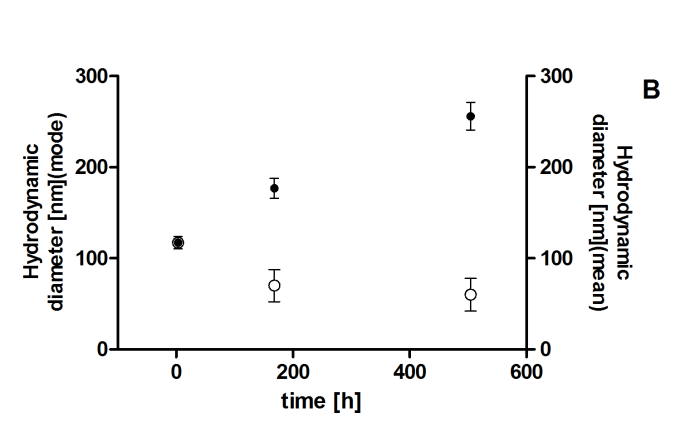

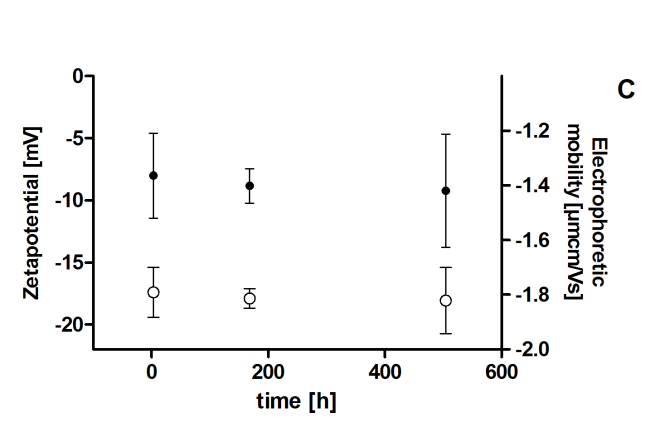

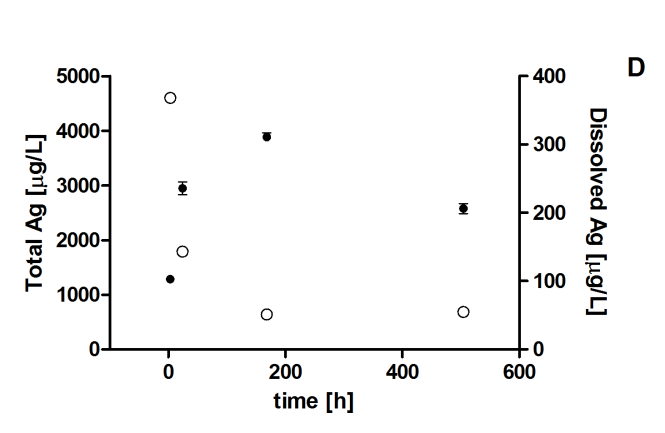


Figure S3: Characteristics of 5 mg/L PVP-Ag NP dispersions in exposure medium stirred at 400 rpm at 15 °C in a 12:12 h light:dark cycle (100 µE/m^2^s). Data is presented as means with standard deviations, n = 3. A: hydrodynamic diameter (determined as zeta-Average; open circles) and Polydispersity index (PDI; closed circles) determined by DLS, B: hydrodynamic diameter (open circles: mode, closed circles: mean) determined by NTA; C: zetapotential (open circles) and electrophoretic mobility (closed circles). D: Total (open circles) and dissolved (closed circles) Ag determined by ICP-MS.


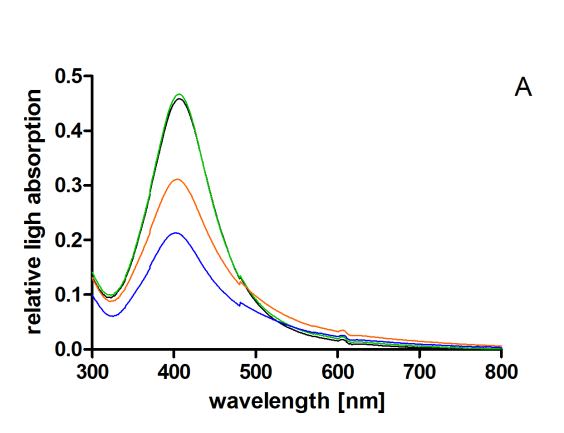

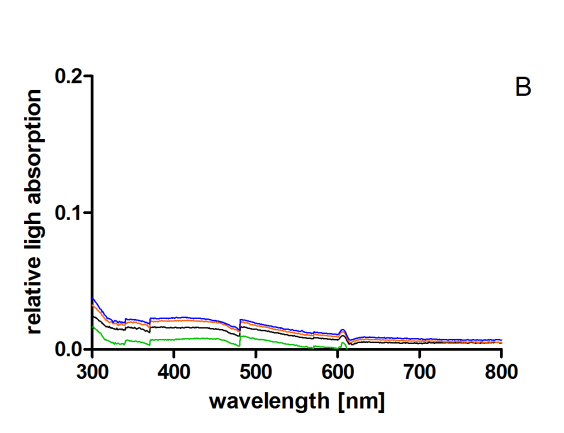

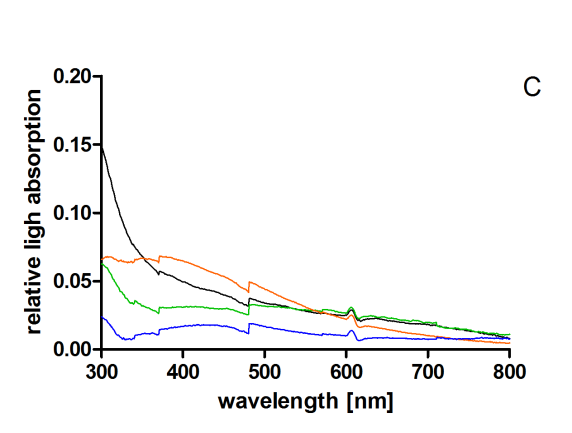

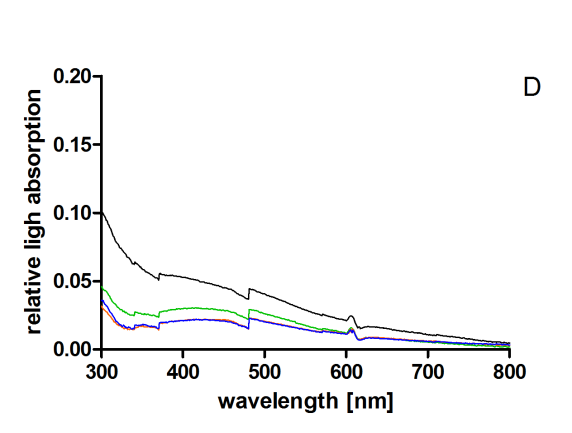

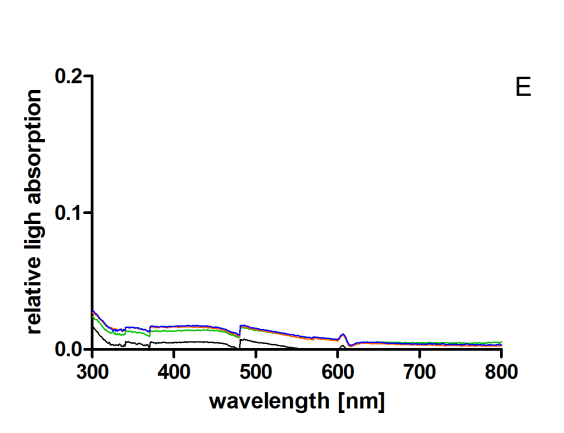


Figure S4: Representative UV-VIS spectra of PVP-Ag NP dispersions and AgNO_3_ stirred in exposure medium at 400 rpm at 15 °C in a 12:12 h light:dark cycle (100 µE/m^2^s). Samples were taken after 3 h (black lines), 24 h (green lines), 168 h/7 d (orange lines), and 504 h/21 d (blue lines). A: 5 mg/L PVP-AgNP, B: 20 µg/L PVP-AgNP, C: 5 mg/L AgNO_3_, D: 20 µg/L AgNO_3_, E: exposure medium.


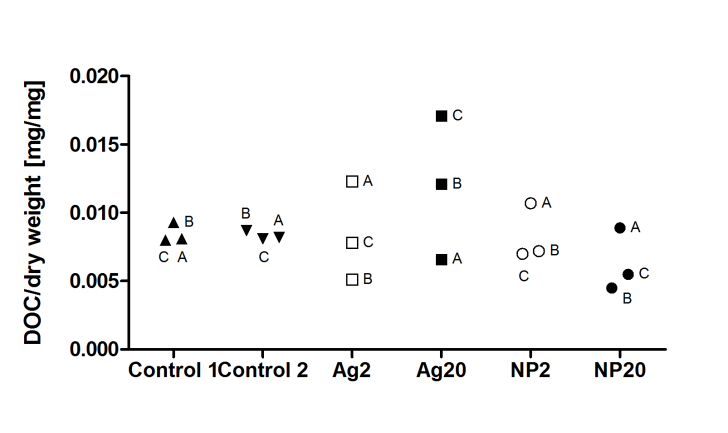


Figure S5: DOC per dry weight of biofilm [mg/mg] on d_18_ of the exposure. The treatment is indicated on the x-axis, data points are labelled by the AIS section samples were collected from. Median DOC per dry weight was not significantly different between the different treatments (Kruskal-Wallis, Dunn’s post-hoc test).


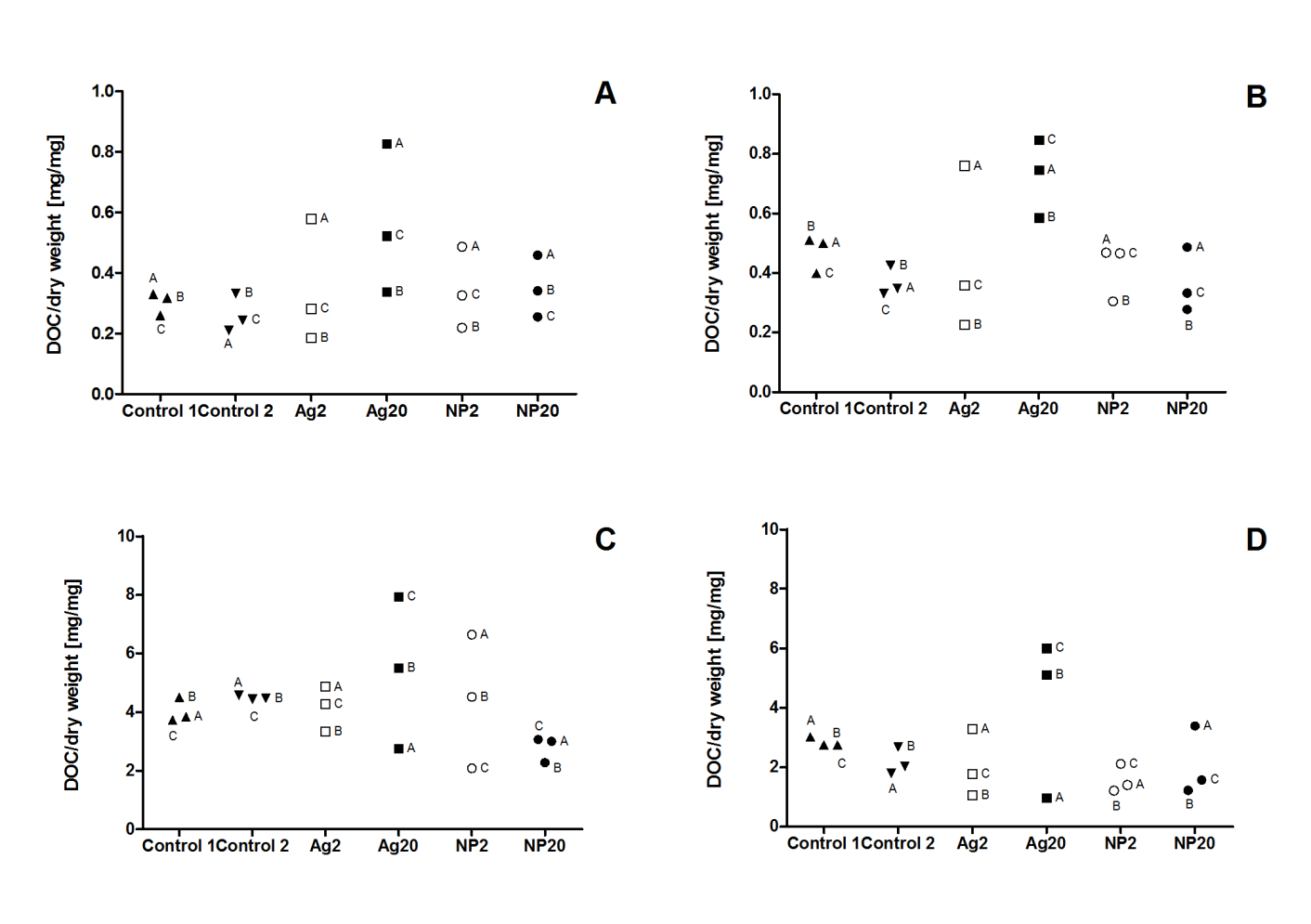


Figure S6: DOC per dry weight of biofilm [mg/mg] on d18 of the exposure corresponding to biopolymers (A), building blocks of humic substances (B), low molecular weight (LMW) acids (C), and amphiphilic/neutral substances (D). The treatment is indicated on the x-axis, data points are labelled by the AIS section samples were collected from. Treatment did not have a significant effect on the fraction of biopolymers, building blocks of HS, low molecular weight (LMW) acids, and amphiphilics/neutrals and there was no interaction between treatment and EPS components (two-way ANOVA, Bonferroni’s post-test, Table S9). Biopolymers and building blocks of HS were always significantly different from LMW acids but only different from amphiphilics/neutrals in samples treated with Ag20 (p<0.001, t_polymers_=5.802, t_building blocks_= 4.094).


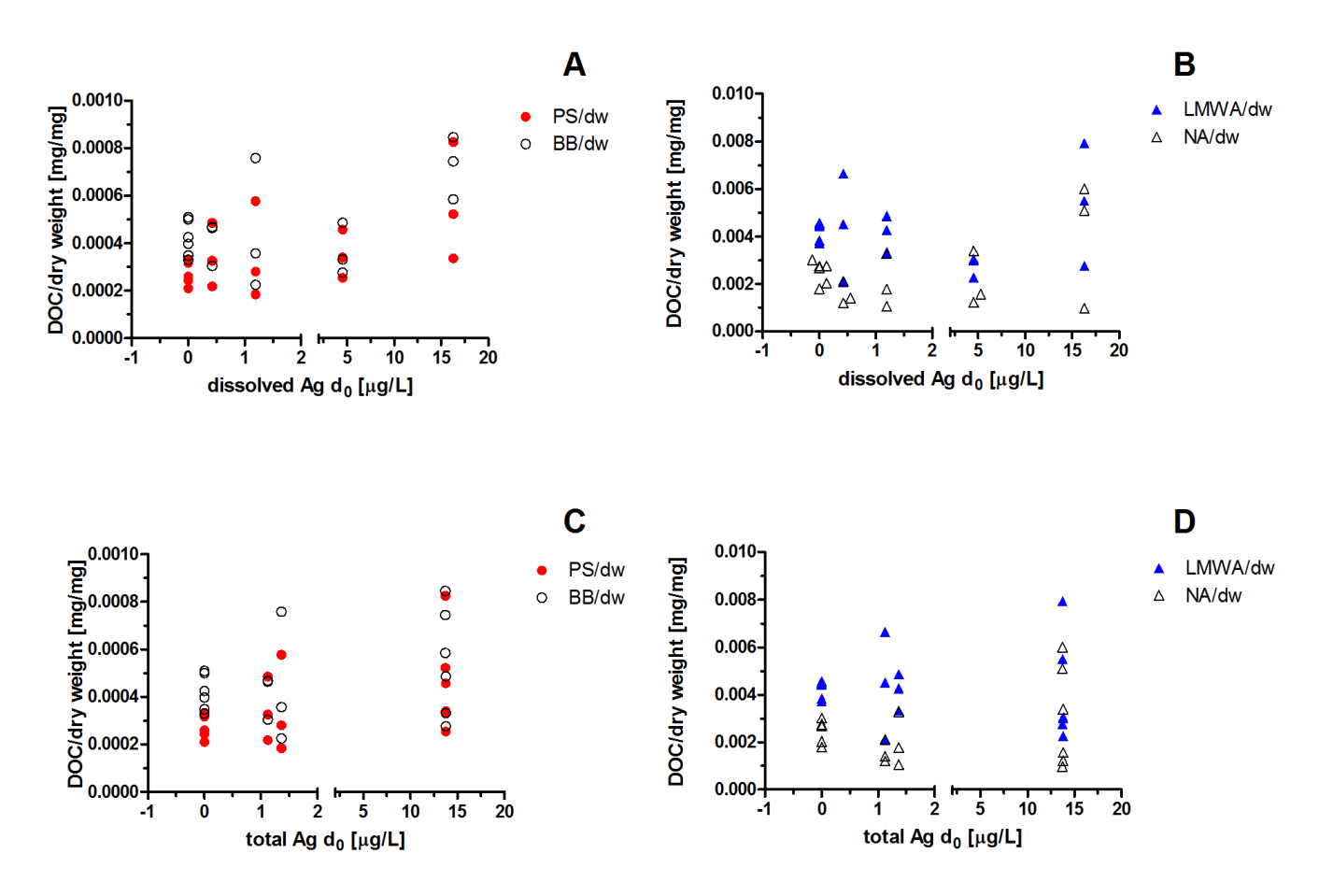


Figure S7: Fraction of EPS components relative to dry weight [mg/mg] plotted against the measured concentration of dissolved (A, B) and total (C, D) Ag. A, C: biopolymers and building blocks; B, D: low molecular weight acids and neutrals/amphiphilics.


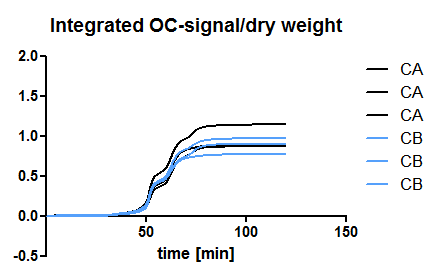

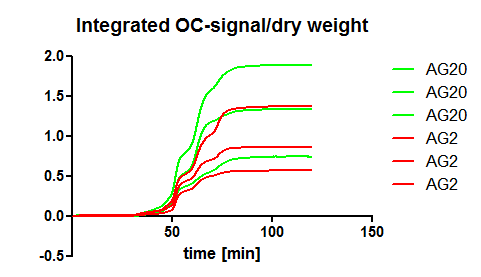

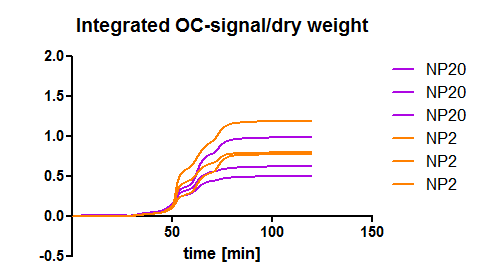

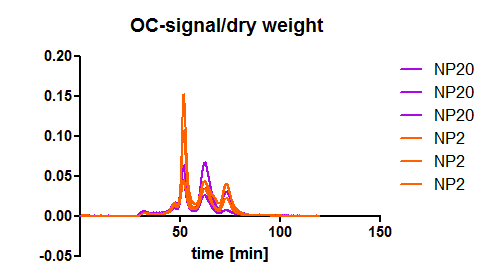

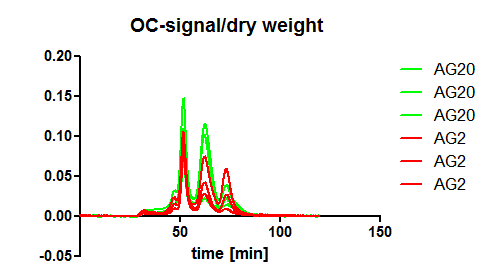

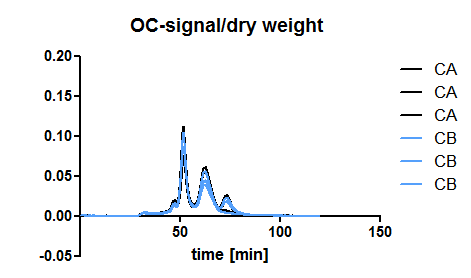


Figure S8: LC-OCD chromatograms (left) and integrals (right) of OC-signals per dry weight [relative intensities and cumulate intensities, respectively, on the y-axes].


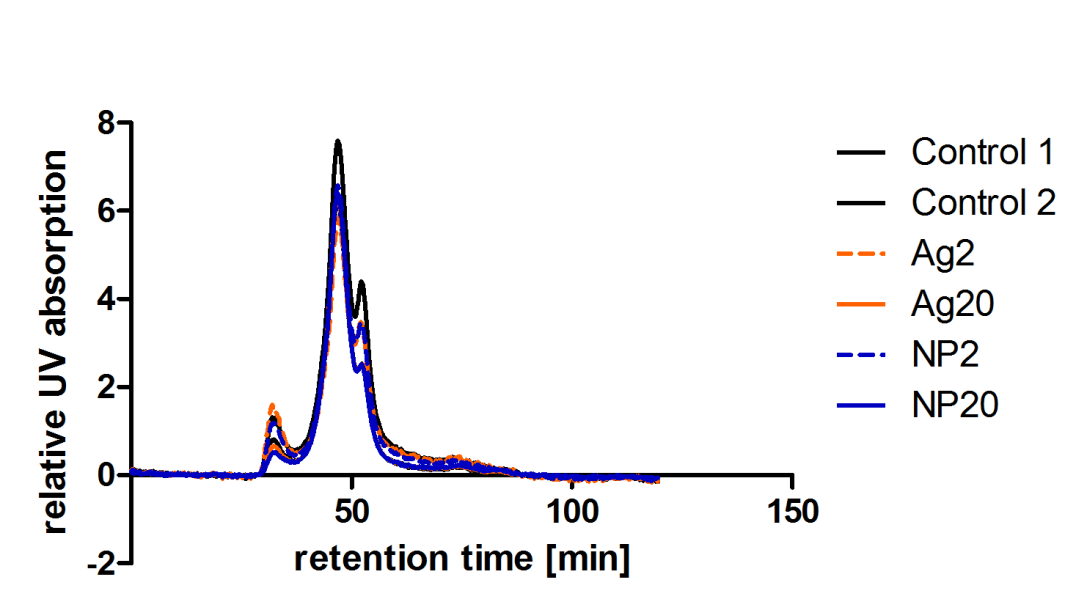


Figure S9: LC-OCD-chromatograms of DOC in the AIS


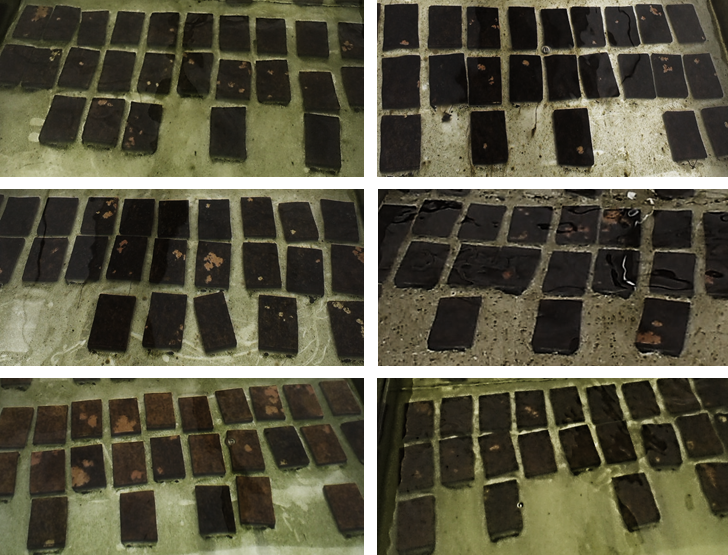


Figure S10: Photographs of the downstream sections of the AIS in the last week of the experiment. Upper row: Control 1 and control 2, middle row: Ag2 and NP2, bottom row: Ag20 and NP20.

**2. Supplementary Tables**

Table S1: Median values of physicochemical parameters over the course of the experiment. Minimum and maximum values are provided in brackets.

|  | Temperature | O_2_ | Conductivity | pH | SRP-P | NO_3_^2-^-N | NO_2_-N | NH_4_^+^-N | NH_3_-N |
| --- | --- | --- | --- | --- | --- | --- | --- | --- | --- |
|  | (°C) | (mg L^-1^) | (µS cm^-1^) |  | (µg L^-1^) | (mg L^-1^) | (mg L^-1^) | (µg L^-1^) | (µg L^-1^) |
| C1 | 15.0 (14.7 - 15.5) | 10.0 (9.5 - 10.3) | 423 (415 - 424) | 8.1 (8.0 - 8.3) | 21.5 (< LOD - 62.6) | 3.7 (1.9 - 3.8) | < LOD | 5.6 (0.8 - 51.4) | 0.2 (0 - 1.6) |
| C2 | 15.4 (14.5 - 15.6) | 9.8 (9.5 - 10.5) | 442 (431 - 446) | 8.0 (7.9 - 8.4) | 30.8 (0.5 - 68.5) | 3.8 (2.4 - 3.8) | < LOD | 15.1 (0.2 - 59.5) | 0.4 (0 - 1.8) |
| Ag 2 | 14.9 (14.3 - 15.3) | 9.8 (9.6 - 10.5) | 441 (436 - 444) | 8.1 (8.0 - 8.3) | 26.1 (< LOD - 68.1) | 3.3 (2.3 - 3.9) | < LOD | 10.8 (3.5 - 62.0) | 0.4 (0.2 - 1.9) |
| Ag 20 | 15.3 (14.4 - 15.6) | 9.7 (9.4 - 10.2) | 440 (437 - 443) | 8.0 (7.9 - 8.3) | 29.7 (0.6 - 72.9) | 3.8 (2.2 - 3.9) | < LOD | 17.1 (2.0 - 59.9) | 0.5 (0.1 - 1.8) |
| NP 2 | 15.1 (14.4 - 15.4) | 10.0 (9.5 - 10.2) | 441 (437 - 442) | 8.1 (8.0 - 8.3) | 10.9 (< LOD - 63.6) | 3.6 (1.8 - 3.9) | < LOD | 9.6 (4.2 - 59.6) | 0.3 (0.2 - 1.8) |
| NP 20 | 15.2 (14.6 - 15.5) | 9.7 (9.5 - 10.6) | 442 (429 - 445) | 8.0 (7.9 - 8.5) | 29.0 (0.3 - 73.2) | 3.7 (2.2 - 3.8) | < LOD | 9.3 (2.7 - 48.5) | 0.4 (0.2 - 1) |

|  | Cl- | F- | Silicate-Si | Ca^2+^ | Mg^2+^ | DOC d_0_ | Humic substances d_0_ |
| --- | --- | --- | --- | --- | --- | --- | --- |
|  | (mg L-1) | (mg L^-1^) | (mg L^-1^) | (mg L^-1^) | (mg L^-1^) | (mg L^-1^) | (mg L^-1^) |
| C1 | 29.70 (28.70-30.60) | 0.1 (0.1-0.1) | 6.1 (5.1 - 7.3) | 25.15 (20.90-31.10) | 11.05 (7.30-11.10) | 2.89 | 1.61 |
| C2 | 29.70 (28.80-30.20) | 0.1 (0.1-0.1) | 5.1 (0.2 - 7.4) | 26.45 (20.80-30.50) | 10.20 (5.50-10.50) | 2.86 | 1.79 |
| Ag 2 | 24.50 (24.30-24.90) | 0.1 (0.1-0.1) | 5.0 (2.7 - 7.6) | 29.25 (27.40-30.70) | 9.80 (5.80-10.60) | 3.05 | 1.76 |
| Ag 20 | 29.60 (29.40-30.20) | 0.1 (0.1-0.1) | 6.2 (5.4 - 6.4) | 28.30 (18.60-30.60) | 10.25 (9.60-11.10) | 2.84 | 1.66 |
| NP 2 | 29.50 (29.00-30.50) | 0.1 (0.1-0.1) | 6.1 (5.2 - 6.6) | 29.25 (18.50-30.30) | 8.85 (6.60-10.60) | 2.79 | 1.58 |
| NP 20 | 29.50 (29.30-30.60) | 0.1 (0.1-0.1) | 4.4 (0.2 - 7.2) | 29.80 (19.30-32.70) | 8.70 (6.20-10.30) | 2.82 | 1.60 |

Table S2: Concentrations [mM] of compounds used as input for VMINTEQ derived from the composition of the exposure media according to Borgmann and the mean tap water composition.

| Species | Concentration [mM] |
| --- | --- |
| Al+3 | 8.52E-04 |
| Ba+2 | 2.77E-04 |
| Br-1 | 1.60E-04 |
| Ca+2 | 1.94E+00 |
| Cd+2 | 2.67E-03 |
| Cl-1 | 9.04E-01 |
| CO3-2 | 1.00E+00 |
| EDTA-4 | 6.72E-03 |
| Fe+2 | 3.58E-03 |
| H3BO3 | 4.62E-02 |
| H4SiO4 | 7.71E-02 |
| I-1 | 2.00E-05 |
| K+1 | 7.84E-02 |
| Li+1 | 2.02E-01 |
| Mg+2 | 4.58E-01 |
| Mn+2 | 2.00E-03 |
| Na+1 | 8.89E-01 |
| NO3-1 | 1.87E-01 |
| SeO4-2 | 1.30E-05 |
| SO4-2 | 6.57E-01 |
| Sr+2 | 7.76E-01 |
| Zn+2 | 1.84E-04 |

Table S3: Results of two-way ANOVA of data plotted in Figure 2

| Source of Variation | % of total variation | P value |  |  |
| --- | --- | --- | --- | --- |
| Interaction | 10.07 | < 0.0001 |  |  |
| Time | 7.45 | < 0.0001 |  |  |
| Row Factor | 67.80 | < 0.0001 |  |  |
|  |  |  |  |  |
| Source of Variation | P value summary | Significant? |  |  |
| Interaction | *** | Yes |  |  |
| Time | *** | Yes |  |  |
| Row Factor | *** | Yes |  |  |
|  |  |  |  |  |
| Source of Variation | Df | Sum-of-squares | Mean square | F |
| Interaction | 15 | 2.298 | 0.1532 | 5.486 |
| Time | 5 | 1.700 | 0.3400 | 12.18 |
| Row Factor | 3 | 15.47 | 5.157 | 184.7 |
| Residual | 120 | 3.351 | 0.02792 |  |
|  |  |  |  |  |
| Number of missing values | 0 |  |  |  |

Table S4: Mass of Ag associated with the biofilms per biomass [µg/g] per sample

| day | Control 1 | | | Control 2 | | | Ag 2 | | | Ag 20 | | | NP 2 | | | NP 20 | | |
| --- | --- | --- | --- | --- | --- | --- | --- | --- | --- | --- | --- | --- | --- | --- | --- | --- | --- | --- |
| 0 | 0.39 | 0.78 | 0.84 | 0.52 | 0.72 | 0.79 | 0.92 | 3.00 | 4.84 | 35.41 | 32.20 | 43.26 | 1.72 | 3.54 | 1.87 | 8.18 | 7.84 | 11.68 |
| 4 | 0.39 | 0.36 | 0.29 | <LOD | 0.37 | 0.72 | 12.81 | 9.94 | 9.50 | 66.33 | 58.02 | 57.52 | 10.46 | 10.76 | 9.46 | 96.58 | 99.77 | 94.00 |
| 11 | <LOD | 0.08 | 0.10 | 0.20 | 0.32 | 0.36 | 2.97 | 2.59 | 2.93 | 20.18 | 20.70 | 19.36 | 5.10 | 5.12 | 5.68 | 40.81 | 29.55 | 39.55 |
| 18 | 0.76 | 0.20 | 0.26 | 0.14 | 0.32 | 0.06 | 2.30 | 1.64 | 1.87 | 14.08 | 15.79 | 17.48 | 4.54 | 3.74 | 4.70 | 25.09 | 23.58 | 24.98 |

Table S5: Semi quantitative species and genera abundance of diatoms and green algae in the six AIS on d_0_ of the experiment. 5: >30%, 4: 30-10%, 3: 10-3%, 2: 3-1%, 1: <1%.

| Treatment | Control 1 | | | Control 2 | | | Ag2 | | | Ag2 | | | NP2 | | | NP2 | | |
| --- | --- | --- | --- | --- | --- | --- | --- | --- | --- | --- | --- | --- | --- | --- | --- | --- | --- | --- |
| AIS section | A | B | C | A | B | C | A | B | C | A | B | C | A | B | C | A | B | C |
| *Achnanthes* |  |  |  |  |  |  |  |  |  |  |  |  |  |  |  |  |  |  |
| *-A. lanceolata* | 1 | 1 | 2 | 1 | 1 | 1 | 1 | 1 | 2 | 2 | 1 | 2 | 1 | 2 | 2 | 1 | 1 | 1 |
| *-A. minutissima* | 2 | 1 | 2 | 1 | 2 | 1 | 2 | 2 | 3 | 2 | 2 | 2 | 1 | 2 | 2 | 2 | 2 | 2 |
| *Amphora pediculus* | 1 | 1 | 1 | 2 | 1 | 2 | 1 | 1 | 1 | 1 | 1 | 1 | 1 | 1 | 1 | 1 | 1 | 1 |
| *Cocconeis placentula* | 2 | 1 | 2 | 2 | 1 | 1 | 1 | 2 | 1 | 2 | 1 | 1 | 2 | 2 | 2 | 1 | 1 | 1 |
| *Gomphonema sp.* | 1 | 2 | 2 | 2 | 1 | 2 | 2 | 2 | 1 | 2 | 2 | 2 | 2 | 1 | 1 | 1 | 2 | 1 |
| *Navicula* |  |  |  |  |  |  |  |  |  |  |  |  |  |  |  |  |  |  |
| *-N. radiosa* | 2 | 2 | 2 | 2 | 2 | 2 | 2 | 2 | 1 | 1 | 2 | 2 | 2 | 1 | 1 | 2 | 2 | 2 |
| *-N. sp.* | 2 | 1 | 2 | 1 | 2 | 1 | 2 | 1 | 2 | 2 | 2 | 2 | 2 | 2 | 2 | 2 | 1 | 1 |
| *Nitzschia* |  |  |  |  |  |  |  |  |  |  |  |  |  |  |  |  |  |  |
| *-N. linearis* | 1 | 1 | 1 | 1 | 1 |  | 1 | 1 | 2 | 1 | 1 | 2 | 1 | 1 | 1 | 1 | 1 | 1 |
| *-N. sp.* | 1 | 1 | 1 | 1 | 1 | 1 | 1 | 1 | 1 | 2 | 2 | 2 | 1 | 1 | 1 | 1 | 1 | 1 |
| *Surirella sp.* | 1 | 1 | 1 | 1 | 1 | 1 | 1 | 1 | 1 | 1 | 1 | 1 | 1 | 1 | 1 | 1 | 1 | 1 |
| *Melosira varians* |  |  |  |  |  |  |  |  |  |  |  |  |  |  | 1 |  |  |  |
| *Rhoicosphenia sp.* |  |  |  |  |  |  |  |  |  |  |  |  |  |  |  |  |  |  |
| *Cymbella affinis* |  |  |  |  |  |  |  |  |  |  |  |  |  |  |  |  |  |  |
| *Fragilaria ulna* |  |  |  |  |  |  |  |  |  |  |  |  |  |  |  |  |  |  |
| *Monodus sp.* | 3 | 3 | 3 | 2 | 3 | 2 | 3 | 3 | 3 | 4 | 3 | 4 | 4 | 4 | 4 | 3 | 3 | 3 |
| *Scenedesmus dimorphus* | 4 | 3 | 3 | 3 | 3 | 3 | 2 | 2 | 3 | 2 | 3 | 2 | 4 | 4 | 4 | 2 | 3 | 2 |
| *Ulothrix sp.* |  |  |  |  |  |  |  |  |  |  |  |  |  |  |  |  |  |  |

Table S6: Semi quantitative species and genera abundance of diatoms and green algae in the six AIS on d_4_ of the experiment. 5: >30%, 4: 30-10%, 3: 10-3%, 2: 3-1%, 1: <1%.

| Treatment | Control 1 | | | Control 2 | | | Ag2 | | | Ag20 | | | NP2 | | | NP20 | | |
| --- | --- | --- | --- | --- | --- | --- | --- | --- | --- | --- | --- | --- | --- | --- | --- | --- | --- | --- |
| AIS section | A | B | C | A | B | C | A | B | C | A | B | C | A | B | C | A | B | C |
| *Achnanthes* |  |  |  |  |  |  |  |  |  |  |  |  |  |  |  |  |  |  |
| *-A. lanceolata* | 3 | 3 | 3 | 3 | 3 | 3 | 2 | 3 | 2 | 2 |  | 1 | 2 | 3 | 4 | 2 | 2 | 2 |
| *-A. minutissima* | 4 | 4 | 4 | 4 | 4 | 4 | 2 | 3 | 3 | 3 | 2 | 3 | 3 | 2 | 3 | 3 | 3 | 4 |
| *Amphora pediculus* | 2 | 1 | 2 | 1 | 2 | 2 | 1 | 2 | 2 |  |  |  | 2 | 2 | 2 | 2 | 2 | 2 |
| *Cocconeis placentula* | 2 | 2 | 3 | 2 | 2 | 2 | 1 | 2 | 1 | 1 | 1 | 1 | 2 | 2 | 2 | 2 | 2 | 2 |
| *Gomphonema sp.* | 2 | 2 | 2 | 2 | 3 | 2 | 3 | 3 | 2 | 1 | 1 | 1 | 2 | 2 | 2 | 3 | 2 | 3 |
| *Navicula* |  |  |  |  |  |  |  |  |  |  |  |  |  |  |  |  |  |  |
| *-N. radiosa* | 3 | 3 | 3 | 3 | 3 | 3 | 3 | 3 | 2 | 2 | 1 | 2 | 3 | 3 | 3 | 3 | 3 | 3 |
| *-N. sp.* | 3 | 3 | 3 | 3 | 3 | 3 | 3 | 3 | 2 | 1 |  | 1 | 3 | 3 | 3 | 3 | 3 | 3 |
| *Nitzschia* |  |  |  |  |  |  |  |  |  |  |  |  |  |  |  |  |  |  |
| *-N. linearis* | 3 | 3 | 4 | 3 | 3 | 3 | 2 | 2 | 2 |  |  |  | 2 | 2 | 2 | 2 | 1 | 2 |
| *-N. sp.* | 2 | 2 | 3 | 3 | 3 | 3 | 2 | 3 | 3 | 3 | 2 | 3 | 2 | 2 | 2 | 3 | 3 | 3 |
| *Surirella sp.* | 1 | 1 | 2 | 2 | 2 | 2 | 1 | 1 | 1 | 1 | 1 | 1 | 1 | 1 | 1 | 1 | 2 | 2 |
| *Melosira varians* |  |  |  |  |  |  |  |  |  |  |  |  |  |  |  |  |  |  |
| *Rhoicosphenia sp.* |  |  |  |  |  |  |  |  |  |  |  |  |  |  |  |  |  |  |
| *Cymbella affinis* |  |  |  |  |  |  |  |  |  |  |  |  |  |  |  |  |  |  |
| *Fragilaria ulna* |  |  |  |  |  |  |  |  |  |  |  |  |  |  |  |  |  |  |
| *Monodus sp.* | 1 | 3 | 1 | 3 | 2 | 2 | 3 | 3 | 3 | 4 | 3 | 3 | 2 | 3 | 3 | 3 | 4 | 4 |
| *Scenedesmus dimorphus* | 5 | 4 | 5 | 4 | 5 | 5 | 3 | 4 | 4 | 3 | 2 | 3 | 5 | 5 | 5 | 2 | 3 | 2 |
| *Ulothrix sp.* | 3 | 2 | 2 | 2 | 2 | 2 | 1 | 1 | 1 | 1 | 1 | 1 | 1 | 1 | 1 | 1 | 1 | 1 |

Table S7: Semi quantitative species and genera abundance of diatoms and green algae in the six AIS on d_18_ of the experiment. 5: >30%, 4: 30-10%, 3: 10-3%, 2: 3-1%, 1: <1%.

| Treatment | Control 1 | | | Control 2 | | | Ag2 | | | Ag20 | | | NP2 | | | NP20 | | |
| --- | --- | --- | --- | --- | --- | --- | --- | --- | --- | --- | --- | --- | --- | --- | --- | --- | --- | --- |
| AIS section | A | B | C | A | B | C | A | B | C | A | B | C | A | B | C | A | B | C |
| *Achnanthes* |  |  |  |  |  |  |  |  |  |  |  |  |  |  |  |  |  |  |
| *-A. lanceolata* | 3 | 3 | 3 | 3 | 3 | 3 | 4 | 4 | 4 | 4 | 4 | 4 | 3 | 3 | 3 | 3 | 4 | 4 |
| *-A. minutissima* | 5 | 5 | 5 | 5 | 5 | 5 | 5 | 5 | 5 | 5 | 5 | 5 | 5 | 5 | 5 | 5 | 5 | 5 |
| *Amphora pediculus* | 3 | 3 | 3 | 4 | 4 | 4 | 3 | 4 | 4 | 1 | 1 | 1 | 4 | 4 | 4 | 4 | 4 | 4 |
| *Cocconeis placentula* | 3 | 3 | 3 | 3 | 3 | 3 | 3 | 3 | 3 | 3 | 3 | 3 | 4 | 4 | 4 | 3 | 3 | 3 |
| *Gomphonema sp.* | 3 | 3 | 3 | 4 | 4 | 4 | 4 | 4 | 4 | 3 | 3 | 3 | 4 | 4 | 4 | 3 | 2 | 3 |
| *Navicula* |  |  |  |  |  |  |  |  |  |  |  |  |  |  |  |  |  |  |
| *-**N. radiosa* | 4 | 3 | 4 | 5 | 4 | 4 | 4 | 4 | 4 | 3 | 3 | 3 | 3 | 4 | 4 | 4 | 4 | 4 |
| *-N. sp.* | 4 | 4 | 4 | 4 | 4 | 4 | 4 | 4 | 4 | 4 | 4 | 4 | 4 | 4 | 4 | 4 | 4 | 4 |
| *Nitzschia* |  |  |  |  |  |  |  |  |  |  |  |  |  |  |  |  |  |  |
| *-N. linearis* | 3 | 3 | 3 | 3 | 3 | 3 | 3 | 3 | 3 | 1 | 1 | 1 | 2 | 3 | 3 | 3 | 3 | 3 |
| *-N. sp.* | 4 | 4 | 4 | 4 | 4 | 4 | 4 | 4 | 4 | 4 | 4 | 4 | 3 | 3 | 3 | 4 | 4 | 4 |
| *Surirella sp.* | 2 | 3 | 3 | 3 | 3 | 3 | 3 | 3 | 3 | 3 | 3 | 3 | 3 | 3 | 3 | 3 | 3 | 3 |
| *Melosira varians* | 1 | 1 | 1 | 2 | 2 | 2 | 1 |  | 1 |  |  |  |  |  |  | 1 |  |  |
| *Rhoicosphenia sp.* | 1 | 2 | 2 | 2 | 2 | 1 | 1 | 2 | 1 |  |  |  | 2 | 2 | 1 | 1 | 1 | 1 |
| *Cymbella affinis* |  |  |  |  |  |  |  | 1 |  |  |  |  |  |  |  |  |  |  |
| *Fragilaria ulna* |  |  |  |  |  |  |  | 1 | 1 |  |  |  |  |  |  |  |  |  |
| *Monodus sp.* |  |  |  |  |  |  |  |  |  | 4 | 3 | 4 |  |  |  |  |  |  |
| *Scenedesmus dimorphus* | 5 | 5 | 5 | 4 | 4 | 4 | 4 | 4 | 5 | 5 | 4 | 4 | 5 | 5 | 5 | 4 | 4 | 4 |
| *Ulothrix sp.* |  |  |  |  |  |  |  |  |  | 1 |  |  |  |  |  |  |  |  |

Table S8: Results of two-way ANOVA on EPS components, Figure S5

| Source of Variation | % of total variation | P value |
| --- | --- | --- |
| Interaction | 4.57 | 0.8006 |
| Time | 4.49 | 0.1050 |
| Column Factor | 72.29 | < 0.0001 |
| Subjects (matching) | 0.3653 | 0.9990 |
|  |  |  |
| Source of Variation | P value summary | Significant? |
| Interaction | ns | No |
| Time | ns | No |
| Column Factor | *** | Yes |
| Subjects (matching) | ns | No |

Table S9: Dissolved and precipitated fractions of components of the exposure medium at equilibrium at 15°C modelled with Vminteq. D: dissolved; P: precipitated; 3 mg/L NOM according to NICA-Donnan was included but did not change the modelled speciation.

|  | 0 µg/L Ag | | | | 20 µg/L Ag | | | | 2 µg/L Ag | | | |
| --- | --- | --- | --- | --- | --- | --- | --- | --- | --- | --- | --- | --- |
|  | D | % D | P | % P | D | % D | P | % P | D | % D | P | % P |
| Ag+1 |  |  |  |  | 1.66E-07 | 89.45 | 1.95E-08 | 10.56 | 7.32E-09 | 39.54 | 1.12E-08 | 60.46 |
| Al+3 | 1.61E-08 | 1.89 | 8.36E-07 | 98.11 | 1.61E-08 | 1.89 | 8.36E-07 | 98.11 | 1.61E-08 | 1.89 | 8.36E-07 | 98.11 |
| Ba+2 | 2.77E-07 | 100.00 | 0.00E+00 | 0.00 | 2.77E-07 | 100.00 | 0.00E+00 | 0.00 | 2.77E-07 | 100.00 | 0.00E+00 | 0.00 |
| Br-1 | 1.60E-07 | 100.00 | 0.00E+00 | 0.00 | 1.60E-07 | 100.00 | 0.00E+00 | 0.00 | 1.60E-07 | 100.00 | 0.00E+00 | 0.00 |
| Ca+2 | 1.63E-03 | 84.33 | 3.04E-04 | 15.67 | 1.63E-03 | 84.33 | 3.04E-04 | 15.67 | 1.63E-03 | 84.33 | 3.04E-04 | 15.67 |
| Cd+2 | 2.67E-06 | 100.00 | 0.00E+00 | 0.00 | 2.67E-06 | 100.00 | 0.00E+00 | 0.00 | 2.67E-06 | 100.00 | 0.00E+00 | 0.00 |
| Cl-1 | 9.04E-04 | 100.00 | 0.00E+00 | 0.00 | 9.04E-04 | 100.00 | 0.00E+00 | 0.00 | 9.04E-04 | 100.00 | 0.00E+00 | 0.00 |
| CO3-2 | 1.57E-04 | 15.71 | 8.43E-04 | 84.29 | 1.57E-04 | 15.71 | 8.43E-04 | 84.29 | 1.57E-04 | 15.71 | 8.43E-04 | 84.29 |
| EDTA-4 | 6.72E-06 | 100.00 | 0.00E+00 | 0.00 | 6.72E-06 | 100.00 | 0.00E+00 | 0.00 | 6.72E-06 | 100.00 | 0.00E+00 | 0.00 |
| Fe+2 | 1.92E-07 | 5.36 | 3.39E-06 | 94.65 | 1.92E-07 | 5.36 | 3.39E-06 | 94.65 | 1.92E-07 | 5.36 | 3.39E-06 | 94.65 |
| H+1 | 1.27E-04 | 100.00 | 0.00E+00 | 0.00 | 1.27E-04 | 100.00 | 0.00E+00 | 0.00 | 1.27E-04 | 100.00 | 0.00E+00 | 0.00 |
| H3BO3 | 4.62E-05 | 100.00 | 0.00E+00 | 0.00 | 4.62E-05 | 100.00 | 0.00E+00 | 0.00 | 4.62E-05 | 100.00 | 0.00E+00 | 0.00 |
| H4SiO4 | 3.58E-05 | 46.39 | 4.13E-05 | 53.61 | 3.58E-05 | 46.39 | 4.13E-05 | 53.61 | 3.58E-05 | 46.39 | 4.13E-05 | 53.61 |
| I-1 | 2.00E-08 | 100.00 | 0.00E+00 | 0.00 | 4.53E-10 | 2.27 | 1.95E-08 | 97.73 | 8.80E-09 | 44.02 | 1.12E-08 | 55.98 |
| K+1 | 7.84E-05 | 100.00 | 0.00E+00 | 0.00 | 7.84E-05 | 100.00 | 0.00E+00 | 0.00 | 7.84E-05 | 100.00 | 0.00E+00 | 0.00 |
| Li+1 | 2.02E-04 | 100.00 | 0.00E+00 | 0.00 | 2.02E-04 | 100.00 | 0.00E+00 | 0.00 | 2.02E-04 | 100.00 | 0.00E+00 | 0.00 |
| Mg+2 | 3.99E-04 | 87.10 | 5.90E-05 | 12.90 | 3.99E-04 | 87.10 | 5.90E-05 | 12.90 | 3.99E-04 | 87.10 | 5.90E-05 | 12.90 |
| Mn+2 | 2.00E-06 | 100.00 | 0.00E+00 | 0.00 | 2.00E-06 | 100.00 | 0.00E+00 | 0.00 | 2.00E-06 | 100.00 | 0.00E+00 | 0.00 |
| Na+1 | 8.89E-04 | 100.00 | 0.00E+00 | 0.00 | 8.89E-04 | 100.00 | 0.00E+00 | 0.00 | 8.89E-04 | 100.00 | 0.00E+00 | 0.00 |
| NO3-1 | 1.87E-04 | 100.00 | 0.00E+00 | 0.00 | 1.87E-04 | 100.00 | 0.00E+00 | 0.00 | 1.87E-04 | 100.00 | 0.00E+00 | 0.00 |
| SeO4-2 | 1.30E-08 | 100.00 | 0.00E+00 | 0.00 | 1.30E-08 | 100.00 | 0.00E+00 | 0.00 | 1.30E-08 | 100.00 | 0.00E+00 | 0.00 |
| SO4-2 | 6.57E-04 | 100.00 | 0.00E+00 | 0.00 | 6.57E-04 | 100.00 | 0.00E+00 | 0.00 | 6.57E-04 | 100.00 | 0.00E+00 | 0.00 |
| Sr+2 | 2.37E-04 | 30.53 | 5.39E-04 | 69.47 | 2.37E-04 | 30.53 | 5.39E-04 | 69.47 | 2.37E-04 | 30.53 | 5.39E-04 | 69.47 |
| Zn+2 | 1.84E-07 | 100.00 | 0.00E+00 | 0.00 | 1.84E-07 | 100.00 | 0.00E+00 | 0.00 | 1.84E-07 | 100.00 | 0.00E+00 | 0.00 |

Table S10: Molar percentage of dissolved Ag^+^ species in the exposure medium at equilibrium at 15°C modelled with Vminteq. 3 mg/L NOM according to NICA-Donnan was included.

| 20 µg/L Ag | | 2 µg/L Ag | |
| --- | --- | --- | --- |
| 33.342 | Ag+1 | 33.03 | Ag+1 |
| 0.021 | AgOH (aq) | 0.021 | AgOH (aq) |
| 61.093 | AgCl (aq) | 60.529 | AgCl (aq) |
| 5.072 | AgCl2- | 5.026 | AgCl2- |
| 0.179 | AgBr (aq) | 0.177 | AgBr (aq) |
| 0.043 | AgI (aq) | 0.968 | AgI (aq) |
| 0.235 | AgSO4- | 0.232 | AgSO4- |

**3. Supplementary Texts**

Text S1: Settings for DLS Measurements

DLS size measurements were performed with a Zetasizer Nano ZS (Malvern Instruments) in DTS1060C cells at 25°C. Refractive indices were 1.828 for Ce and 0.45 for Ag, the dispersant was set to water. Further settings were: 173°C backscatter, automatic measurement duration, 3 measurements per sample, automatic attenuation, and automatic optimum measurement position. Data was processed in general purpose mode. Settings for electrophoretic mobility/zetapotential measurements were automatic attenuation and voltage, a minimum of 10 runs, and a maximum of 30 runs.

Text S2: Settings for NTA Measurements

Nanoparticle tracking analysis (NTA, NanoSight LM10 equipped with a LM14 temperature controller (NanoSight Ltd.)) was used to determine a number based particle size distribution. Settings were as follows:

Background Extract: On, Brightness: 0, Gain: 1, Blur Size: 9x9, Detection Threshold Type: Single, Detection Threshold: 15, Min track length 10, Min Expected Size: Auto, Temperature: 23 °C, Viscosity: 0.9326

Text S3: Procedure used to model surfaces in Imaris

Surfaces based on absolute intensity of FITC and chlorophyll fluorescence as well as reflected laser light were created sequentially using manual thresholding. Surfaces were merged and all values exported to *.csv.
